# CRISPR/Cas9 editing of three CRUCIFERIN C homoeologues alters the seed protein profile in *Camelina sativa*

**DOI:** 10.1186/s12870-019-1873-0

**Published:** 2019-07-04

**Authors:** Wendy J. Lyzenga, Myrtle Harrington, Diana Bekkaoui, Merek Wigness, Dwayne D. Hegedus, Kevin L. Rozwadowski

**Affiliations:** 1 Present address: Global Institute for Food Security, University of Saskatchewan, Saskatoon, SK S7N 4J8 Canada; 2 Agriculture and Agri-Food Canada, 107 Science Place, Saskatoon, SK S7N 0X2 Canada; 3 Department of Food and Bioproduct Sciences, University of Saskatchewan, Saskatoon, SK S7N 5A8 Canada

**Keywords:** CRISPR/Cas9, Camelina, Gene editing, Mutation detection, Drop-off assay, Droplet digital PCR, Seed storage protein, Cruciferin, Proteome rebalancing, Amino acid content, Fatty acid profile

## Abstract

**Background:**

The oilseed *Camelina sativa* is grown for a range of applications, including for biofuel, biolubricants, and as a source of omega-3 fatty acids for the aquaculture feed industry. The seed meal co-product is used as a source of protein for animal feed; however, the low value of the meal hinders profitability and more widespread application of camelina. The nutritional quality of the seed meal is largely determined by the abundance of specific seed storage proteins and their amino acid composition. Manipulation of seed storage proteins has been shown to be an effective means for either adjustment of nutritional content of seeds or for enhancing accumulation of high-value recombinant proteins in seeds.

**Results:**

CRISPR/Cas9 gene editing technology was used to generate deletions in the first exon of the three homoeologous genes encoding the seed storage protein CRUCIFERIN C (*CsCRUC*), creating an identical premature stop-codon in each and resulting in a *CsCRUC* knockout line. The mutant alleles were detected by applying a droplet digital PCR drop-off assay. The quantitative nature of this technique is particularly valuable when applied to polyploid species because it can accurately determine the number of mutated alleles in a gene family. Loss of CRUC protein did not alter total seed protein content; however, the abundance of other cruciferin isoforms and other seed storage proteins was altered. Consequently, seed amino acid content was significantly changed with an increase in the proportion of alanine, cysteine and proline, and decrease of isoleucine, tyrosine and valine. *CsCRUC* knockout seeds did not have changed total oil content, but the fatty acid profile was significantly altered with increased relative abundance of all saturated fatty acids.

**Conclusions:**

This study demonstrates the plasticity of the camelina seed proteome and establishes a CRUC-devoid line, providing a framework for modifying camelina seed protein composition. The results also illustrate a possible link between the composition of the seed proteome and fatty acid profile.

**Electronic supplementary material:**

The online version of this article (10.1186/s12870-019-1873-0) contains supplementary material, which is available to authorized users.

## Background

*Camelina sativa* (camelina) is an under-exploited Brassicaceae oilseed crop that has received renewed interest due to a favourable blend of agronomic properties, a high seed oil content (30–49%) with unique composition, and amenability to genetic manipulation and improvement [1]. Initial efforts in camelina were driven by its potential as a biodiesel feedstock requiring low-input for production [2, 3]. Accordingly, the fatty acid profile of camelina seed oil has been engineered for elevated oleic acid content [4–6]. In addition, the high omega-3 (alpha-linolenic acid, 30–43%) content of camelina oil holds potential as a terrestrial replacement for fish oil in aquaculture [1, 7–9]. Correspondingly, camelina has been used for the production of the omega-3 fish oils eicosapentaenoic acid and docosahexaenoic acid [10, 11]. Similar to other oilseed crops, the seed meal remaining after oil extraction is considered a co-product. Camelina seed protein content (20–29%) results in a rich meal that combined with its relatively low levels of glucosinolates makes it attractive for livestock and aquaculture feed [1]. However, tailoring of the quality and content of the seed meal is important for improving the economic competitiveness of camelina oil and thereby increasing the sustainability and profitability of camelina production and processing [12–15].

Seed meal is largely composed of seed storage proteins that serve as a nutrient resource during early seedling development [16]. During seed maturation, carbon and nitrogen resources are largely directed toward production of these highly abundant proteins [16, 17]. The relative abundance and amino acid content of different seed storage proteins influence the nutritional quality and economic value of the seed meal [13]. Manipulation of seed storage proteins is an area of interest in a number of plant species for improvement of nutrient composition and to express foreign proteins [15, 18–22]. Such efforts are largely constrained by the inherent metabolic programming directing production of endogenous seed storage proteins [17, 19]. Reduction of seed storage proteins using gene knock-out or knock-down approaches has been effective in by-passing these limits and increasing foreign protein yield in soybean [19], *Arabidopsis* [23] and rice [21, 24] by making available metabolic resources originally monopolized by endogenous seed storage protein synthesis.

The cruciferins (legumin-type globulins; 11S or 12S) and napins (napin-type albumins; 2S) are the predominant classes of seed storage proteins in the Brassicaceae [13]. In camelina, 60% of seed meal protein is composed of cruciferins [25], thus altering cruciferin abundance is a key target for modulating meal protein composition. The genome sequence of *C. sativa* [26] possesses 12 genes encoding cruciferins that are classified into four families (A, B, C, and D) [27]. In this study, a *CsCRUC* knockout line was generated using CRISPR/Cas9 gene editing to provide a platform for investigating CRUC effects on the camelina seed proteome.

CRISPR/Cas9 is rapidly transforming genetic studies in crop species because of its specificity, ease of use, and ability to generate novel alleles [28, 29]. In this system, the Cas9 endonuclease binds to a single guide RNA (gRNA), which directs the complex to the genomic target locus with homology to the 20 base pair programmable spacer region of the gRNA [30]. The resulting DNA double-stranded break may be repaired through non-homologous end joining (NHEJ) or homology-directed repair (HDR) mechanisms. Error-prone repair through NHEJ tends to generate insertions or deletions (indels) at the targeted locus, which can alter the reading frame and create functionally null knockout alleles through formation of non-sense mutations leading to truncated and non-functional proteins. HDR has the potential to make prescribed substitutions and generate specific edits based on the sequence of the repair template [31]. CRISPR/Cas9 gene editing has been deployed in a variety of plant species and, recently, in camelina [5, 6, 32].

Similar to many crops, *C. sativa* is polyploid and is composed of three highly undifferentiated sub-genomes [26]. Often, mutations in each homoeologue from all three sub-genomes (G1, G2, G3), totalling six mutated homoeoalleles, are required to manifest a phenotype; in other cases where gene dosage is a factor, mutations in only one or two homoeoalleles may be necessary [5]. Conventional methods for detecting CRISPR/Cas9-mediated mutations are not well-suited for polyploids as these methods are not quantitative. In this study, a droplet digital PCR (ddPCR) method [33, 34] was applied to detect heritably stable progenitor mutations and to precisely determine the number of CRISPR/Cas9-mediated mutant alleles and wild-type alleles present in camelina lines. Using this ddPCR drop-off assay, a complete *CsCRUC* knockout line was identified with all six homoeoalleles mutated. Consistent with the proteome rebalancing theory [17], the loss of CRUC did not alter total seed protein content, but did alter the composition of the seed protein profile and levels of some amino acids, as well as several fatty acids. This *CsCRUC* knockout line provides a means to assess the effects of loss of CRUC on the seed proteome and seed composition, and also provides a platform for investigating the directed manipulation of seed meal protein composition.

## Results

### Design of the CsCRUC gRNA spacer sequence and CRISPR/Cas9 construct

**Fig. 1:**
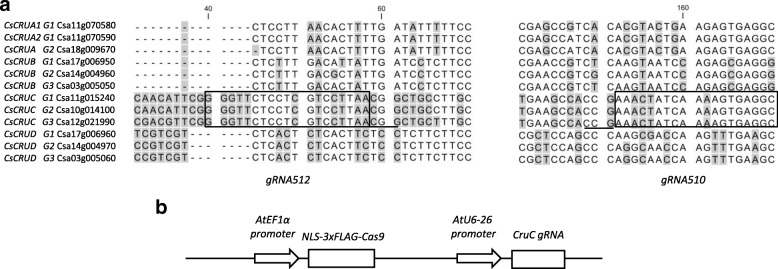
Specificity of *CsCRUC* gRNA spacer design and schematic of CRISPR/Cas9 construct. **a** Partial sequence alignment of the first exon from *CsCRUA, CsCRUB, CsCRUC*, and *CsCRUD* gene families. The *CsCRUC* gRNA spacer sequences (gRNA512 and gRNA510) used in this study are boxed with the PAM sequence underlined. Numbers indicate nucleotide position from the start codon and shading indicates differences in nucleotide sequence. **b** Schematic of the CRIPSR/Cas9 construct. Expression of *Cas9* is under control of the *AtEF1α* promoter and encodes a nuclear localization signal (NLS) at the N- and C-termini, plus a 3xFLAG epitope tag. The *AtU6–26* promoter drives expression of the *CsCRUC* sgRNA cassette. Construct is not represented to scale

The *C. sativa* genome sequence encodes three homoeologues of *CRUCIFERIN C* (*CRUC*), which correspond to its three sub-genomes (*CsCRUC-G1, CsCRUC-G2,* and *CsCRUC-G3;* for gene identifiers see Fig. 1a) [26]. Similarly, the *C. sativa* genome contains three homoeologues of *CRUCIFERIN B* (*CsCRUB-G1, CsCRUB-G2,* and *CsCRUB-G3*) with directly adjacent homoeologues of *CRUCIFERIN D* (*CsCRUD-G1, CsCRUD-G2,* and *CsCRUD-G3*), as found in *Arabidopsis thaliana* [35–37]. Three genes also encode CRUCIFERIN A: two paralogues on G1 (*CsCRUA1-G1, CsCRUA2-G1*)*,* and one homoeologue on G3 (*CsCRUA-G3*); there is no G2 member. This study focused on the *CRUC* homoeologues as this group has the most abundant transcript of the gene family [38] (Additional file 1: Figure S1), as observed in *Arabidopsis* [36], and is the most divergent at the amino acid level [37, 39], thereby making elimination of CRUC a good target for altering camelina seed protein and amino acid composition. Additionally, CRUC has unique structural and physico-chemical properties, and reduced bioaccessibility [39, 40]; thus, its elimination may significantly affect camelina seed meal quality and utility.

The sequence of the first exon of the *CRUCIFERIN* genes was aligned and searched for potential gRNA spacer sequences that would be specific to *CsCRUC-G1, CsCRUC-G2,* and *CsCRUC-G3*, based upon uniqueness of the requisite protospacer adjacent motif (PAM) sequence and mismatches or indels vs. the other cruciferin genes (Fig. 1a). A gRNA spacer sequence (gRNA-512) that anneals to the transcribed strand and one gRNA spacer sequence (gRNA-510) targeting the non-transcribed strand (Fig. 1a) were selected. An *Arabidopsis* RNA polymerase III-dependent promoter derived from *AtU6–26* encoding a member of the spliceosome complex was used for expressing the gRNA cassette (Fig. 1b; Additional file 13 [41]). Due to its high expression in rapidly growing tissues, including meristems and developing gametophytes, the *Arabidopsis EF1α* promoter [42] was used to express *Cas9* codon optimised for expression in crucifers (Additional file 14). Transgenic lines with gRNA512 or gRNA510 and *Cas9* were generated by floral dip [43], and screened for transformants using glufosinate herbicide selection. T_1_ lines were selected for further evaluation based on expression of the *Cas9* transgene (gRNA512-line 1, gRNA512-line 13, gRNA510-line 7 and gRNA510-line 9).

### Design and validation of droplet digital PCR drop-off assay

**Fig. 2:**
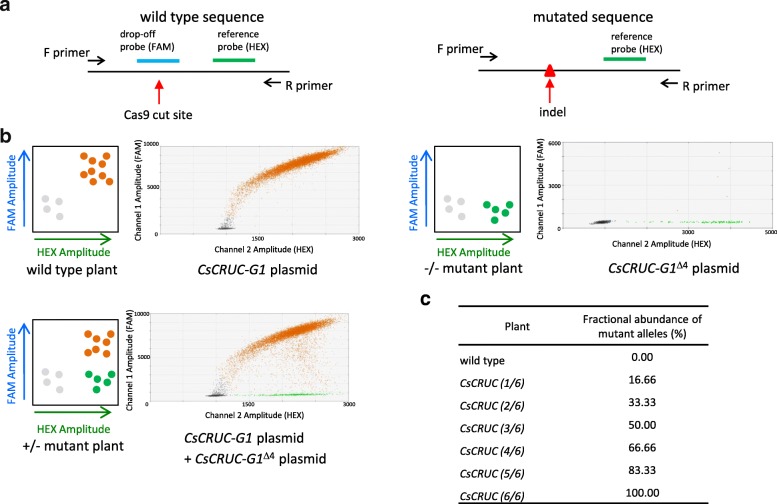
Overview and validation of ddPCR drop-off assay to detect CRISPR/Cas9-mediated mutations. **a** Schematic of probe and primer configuration for detection of mutations. Both the drop-off probe (FAM) and reference probe (HEX) bind to the same amplicons derived from wild-type sequences. Sequence polymorphisms prevent binding of the drop-off probe and only the reference probe binds to amplicons with mutations at the Cas9 cut site. **b** Schematic of anticipated drop-off assay outcome represented on a 2-D fluorescence intensity plot and results using synthesized *CsCRUC-G1* plasmid template. Wild type plants or *CsCRUC-G1* plasmid generate amplicons in which both drop-off probe and reference probe bind, generating only double-positive (HEX and FAM) droplets. Mutant plants with no wild-type sequence or *CsCRUC-G1*^Δ4^ plasmid containing a four base pair deletion at the predicted Cas9 cut site generate amplicons in which only the reference probe binds, resulting in only single-positive (HEX) droplets. Plants with wild-type sequence and mutated sequence or a mixture of *CsCRUC-G1* plasmid and *CsCRUC-G1*^Δ4^ plasmid generate wild-type amplicons in which both drop-off probe and reference probe bind, generating double-positive droplets, and mutation-containing amplicons in which only the reference probe binds, generating single-positive droplets. **c** Expected fractional abundance of drop-off probe events vs. reference probe events representing when heritable mutations have occurred in one to six alleles of *CsCRUC*

A ddPCR [44] drop-off assay [33, 34] was applied to detect CRISPR/Cas9-mediated mutations in *CsCRUC*. A duplex primer probe assay was designed consisting of a drop-off probe (FAM fluorophore) that binds only to the wild-type gRNA annealing site and a reference probe (HEX fluorophore) that binds 150–200 base pairs away from the predicted Cas9 cut site (Fig. 2a). With wild-type DNA both probes will bind resulting in double-positive droplets presenting both fluorescent signals (Fig. 2b). In the event of a CRISPR/Cas9-mediated mutation the drop-off probe (FAM) will no longer bind, resulting in single-positive (HEX) droplets indicative of a mutated allele (Fig. 2b). Because ddPCR is an absolute quantification assay, the number of wild-type and mutated allele sequences can be determined based on the fractional abundance of reference probe events and drop-off probe events (Fig. 2c).

Probe and primer assays for the predicted Cas9 cut sites based on both gRNA510 and gRNA512 were validated using synthesized DNA cassettes containing either wild type *CsCRUC* sequence or a 4-base deletion (*CsCRUC*^*Δ4*^) at the predicted Cas9 cut site (Fig. 2b and Additional file 2: Figure S2). As expected, wild type *CsCRUC* generated double-positive droplets with signals for both the drop-off probe and reference probes, whereas *CsCRUC*^*Δ4*^ generated single-positive droplets with signal for only the reference probe. A mixture of both *CsCRUC* and *CsCRUC*^*Δ4*^ casssettes generated both double-positive and single-positive droplets. As predicted, no droplets were positive for only the drop-off probe.

### Detection of CRISPR/Cas9 mediated mutations in *CsCRUC* in the T_2_ generation

**Fig. 3:**
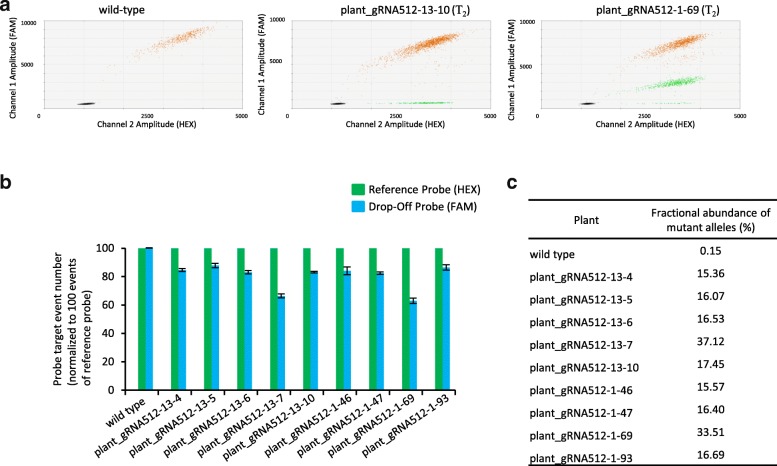
Detection of *CsCRUC* alleles with CRISPR-mediated mutations in T_2_ generation plant lines using a ddPCR drop-off assay. **a** Fluorescence intensity plot from wild type camelina displaying double-positive droplets for both the drop-off probe (FAM) and reference probe (HEX). Plant_gRNA512–13-10 and plant_gRNA512–1-69 are representative lines containing CRISPR-mediated mutations and generate droplets that are double-positive (FAM and HEX) and droplets that are single-positive (HEX). **b** Quantification of drop-off probe events and reference probe events in wild type and nine T_2_ plants containing mutations. Probe targets are normalized to 100 reference probe events. Values are an average of three replicates ± S.D. Differences between expected reference probe events and observed reference probe events were assessed using a chi-square test. **c** Fractional abundance (%) of mutated alleles from b

To identify plant lines with heritable mutations in *CsCRUC* we screened the T_2_ generation instead of the T_1_ generation. The ddPCR drop-off analysis using wild-type genomic DNA solely displayed the expected double-positive signals (Fig. 3a). Twenty of the 85 T_2_ plants evaluated from gRNA512–1 and 11 of the 85 T_2_ plants from gRNA512–13 displayed single-positive droplets, indicative of mutations at the predicted Cas9 cut site (Fig. 3a and Table 1). The drop-off assay enabled estimation of the number of mutant alleles in these plants (Fig. 3b, c). The majority contained only one mutated allele (~ 16% fractional abundance of mutant alleles). Two plants were detected with two mutated alleles (~ 33% fractional abundance of mutant alleles); these plants were designated gRNA512–1-69 and gRNA512–13-7 (Fig. 3a; Table 1). Screening of 90 T_2_ progeny plants from each of gRNA510–7 and gRNA510–9 failed to detect any plants with mutations; material from these lines was not analysed further. To verify the drop-off assay results and to investigate the nature of mutations generated by gRNA512, *CsCRUC* gene sequences were amplified by PCR, cloned and sequenced from four gRNA512–1 and five gRNA512–13 derived T_2_ plants identified as possessing mutations by the drop-off assay. Approximately 30 cloned amplicons per plant were sequenced (Additional file 3: Figure S3). The majority of mutations across plant lines were single base-pair deletions (Table 1). Consistent with the results from the ddPCR drop-off assay, the plants identified with two mutated alleles (gRNA512–1-69 and gRNA512–13-7) in the initial population screen were confirmed to contain two mutant *CsCRUC* alleles (Table 1 and Additional file 3: Figure S3). Line gRNA512–1-69 contains an identical single base pair deletion in both a *CsCRUC-G1* and *CsCRUC-G3* allele (gRNA512–1-69^wt/− 1, wt/wt, wt/− 1^) and was selected for further investigation.

**Table 1:**
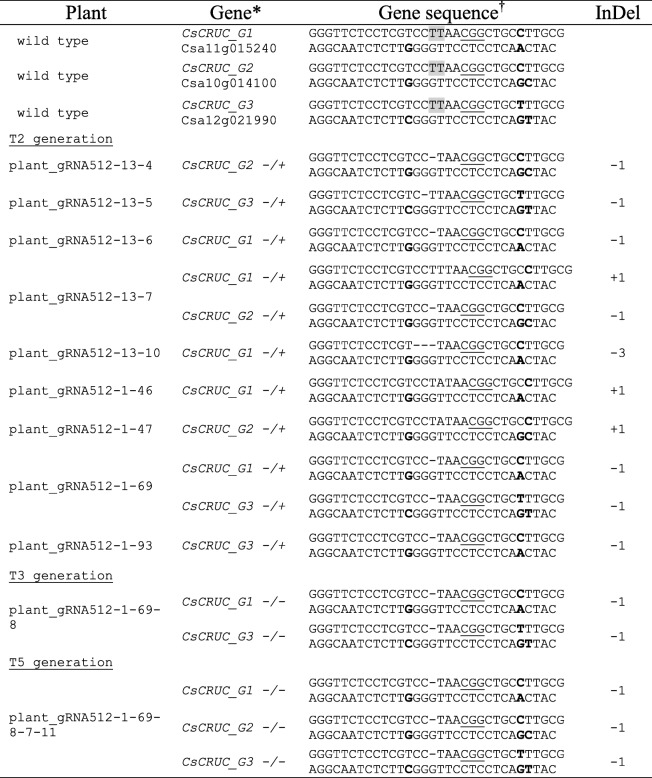
*CsCRUC* allele sequences

*Zygosity of plant at *CsCRUC* homoeologues is indicated with *+* and *-*

^†^The predicted Cas9 cut site is highlighted in grey. PAM sequences are underlined and SNPs that distinguish homoeologues are bolded. Nucleotide changes (+, insertion; –, deletion) are indicated.

### Screening for CRISPR/Cas9 mediated mutations in CsCRUC in the T_3_, T_4_, and T_5_ generation

**Fig. 4:**
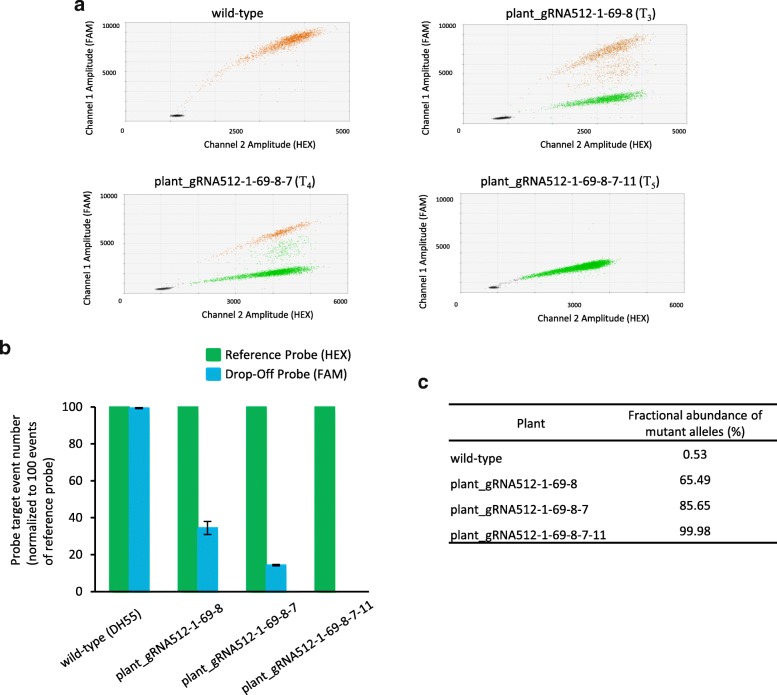
Detection of *CsCRUC* alleles with CRISPR/Cas9-mediated mutations in the T_3_, T_4_, and T_5_ generations using a ddPCR drop-off assay. **a** Fluorescence intensity plots of drop-off assays representing occurrence of single- and double-positive droplets for both the drop-off probe (FAM) and reference probe (HEX). Plant_gRNA512–1–69-8, plant_gRNA512–1–69-8-7, and plant_gRNA512–1–69-8-7-11 are from the T_3_, T_4_ and T_5_ generation, respectively. **b** Quantification of drop-off probe events and reference probe events from a. Probe targets are normalized to 100 reference probe events. Values are an average of three replicates ± S.D. Differences between expected reference probe events and observed reference probe events were tested using a chi-square test. **c** Fractional abundance (%) of mutated alleles from b. Plant_gRNA512–1–69-1-8, plant_gRNA512–1–69-8-7, and plant_gRNA512–1–69-8-7-11 contain 4, 5 and 6 mutated alleles, respectively

The gRNA512–1-69^wt/− 1, wt/wt, wt/− 1^ line retained the CRISPR/Cas9 transgene, as determined by detecting *Cas9* using PCR, and was propagated to the T_5_ generation in order to identify a line with six mutated *CRUC* alleles. Using the drop-off assay we screened T_3_, T_4_ and T_5_ generation plants and identified lines gRNA512–1–69-8 (4/6 mutated alleles), gRNA512–1–69-8-7 (5/6 mutated alleles), and gRNA512–1–69-8-7 − 11 (6/6 mutated alleles), respectively (Fig. 4). The *CsCRUC* alleles in representative lines were amplified by PCR, cloned and sequenced, revealing an identical single base pair deletion in the mutated *CsCRUC* alleles (Table 1, Fig. S3). The genotype gRNA512–1–69-8-7 − 11^–1/− 1, − 1/− 1, − 1/− 1^ is hereafter referred to as *CsCRUC*^−/−,−/−,−/−^.

### Analysis of protein and amino acid profile of CsCRUC knockout seeds

The *CsCRUC*^−/−,−/−,−/−^ line contains a single base pair deletion at nucleotide 53 of the open reading frame of each homoeologue generating a premature stop codon at codon 42 (Additional file 7: Table S1). Under the same growing conditions, wild-type and *CsCRUC*^−/−,−/−,−/−^ plants did not exhibit noticeable differences in growth and development. Seed weight was found not to significantly differ between the genotypes (Table 2). For seed protein extracts, neither the soluble protein content of wild-type and *CsCRUC*^−/−,−/−,−/−^, as determined using a Qubit assay, nor the total protein content, as determined by nitrogen content analysis (% N) of defatted seed meal, was found to be significantly different (Table 2). These results demonstrate inactivation of *CsCRUC* does not impact overall protein content and suggests that abundance of other seed storage proteins is likely elevated to maintain overall protein content in the *CsCRUC*^−/−,−/−,−/−^ seeds.

**Table 2:** Knockout of *CsCRUC* does not alter seed weight, protein or oil content

Line	100 seed weight (mg)^a^	protein content (mg/g dry seed)^b^	protein content of defatted meal (% N based)^b^	oil content (mg/g dry seed)^c^
wild type	114.3 ± 1.7	289.24 ± 9.30	44.01 ± 0.29	355.33 ± 5.08
*CsCRUC* ^−/−,−/−,−/−^	105.7 ± 4.9	306.45 ± 30.24	44.48 ± 0.05	364.40 ± 4.31

^a^Seed weight means are from three samples of seed from each of eight biological replicates

^b^Protein analysis means are from three samples of seed from each of three biological replicates

^c^Oil analysis means are from one sample of seed from each of five biological replicates

^a-c^Means of wild-type and *CsCRUC*^−/−,−/−,−/−^ are not significantly different (*p* < 0.05, Student’s t-test). Means are shown ± S.E.M

**Fig. 5:**
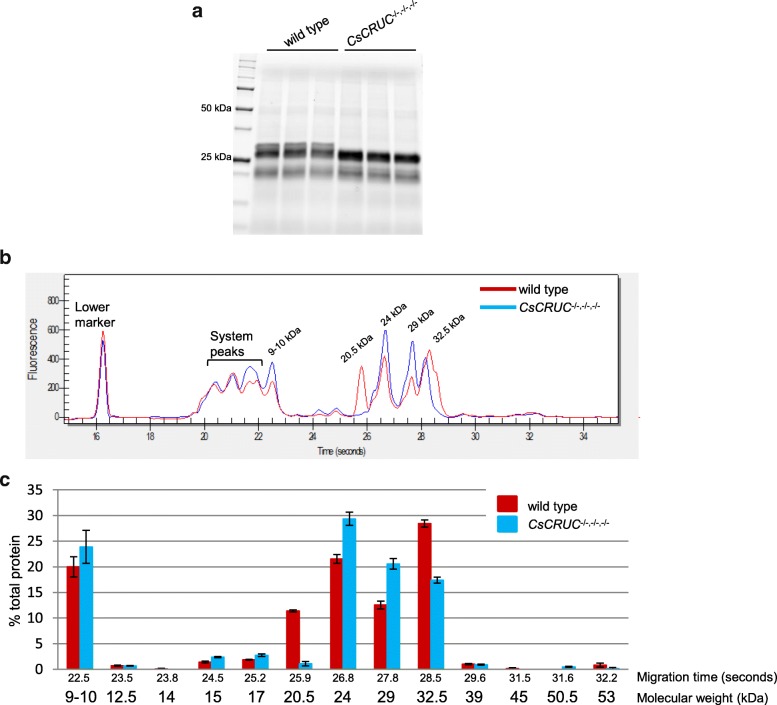
Loss of CRUC in camelina is compensated by accumulation of other seed proteins. **a** Seed protein extracts of wild type and *CsCRUC*^−/−,−/−,−/−^ electrophoresed on 8–16% acrylamide gels under reducing conditions. Gels display three biological replications. **b** Representative electropherogram from microfluidic electrophoresis of wild type and *CsCRUC*^−/−,−/−,−/−^ seed protein extract under reducing conditions. Molecular weight of protein peaks are assigned based on a standard curve. **c** Quantification of b based on corrected peak area calculated by Experion software. Data in c represents the mean ± S.E.M of triplicate measurements from each of three biological replicates

Cruciferins are synthesized as preproteins that are cleaved into α and β chains linked by a disulfide bond to form protomers, three of which combine into the final hexameric structures [13]. SDS-PAGE analysis of seed protein extracts under non-reducing conditions typically display an abundant 48–56 kDa band, characteristic of cruciferin protomers that resolve under reducing conditions as a cluster of α chain (29–34 kDa) and β chain (20–23 kDa) protein bands [13, 25, 39]. Tris-Glycine-extended (TGX)-gel analysis was used to determine if the pattern of soluble protein content was altered in *CsCRUC*^−/−,−/−,−/−^ seeds. Both the wild-type and the *CsCRUC*^−/−,−/−,−/−^ seed protein extracts displayed the expected cluster of α and β chain protein bands under reducing conditions (Fig. 5a) and the characteristic protomer band under non-reducing conditions (Additional file 4: Figure S4a). However, under reducing conditions the *CsCRUC*^−/−,−/−,−/−^ seed protein extract was distinct from wild type by the absence of the highest molecular weight band. This pattern of proteins is consistent with inactivation of *CsCRUC* by the CRISPR/Cas9-derived nonsense mutation since the CRUC α chain is the largest among the cruciferins with a predicted molecular weight of ~ 31.5 kDa being 3–4 kDa greater than the other α chains (Additional file 8: Table S2), and is absent in the knockout line.

To further quantify and characterize the protein pattern of the *CsCRUC*^−/−,−/−,−/−^ line the Experion chip-based microfluidics electrophoresis system was used [45]. The Experion software integrates signals detected for internal standard markers and sample proteins and displays the data as an electropherogram with protein abundance being quantifiable based on peak area; this system has been used to evaluate wheat seed storage proteins [46, 47]. Similar to the TGX-gel analysis, the results from microfluidics electrophoresis indicated the protein profile of *CsCRUC*^−/−,−/−,−/−^ seeds was altered compared to wild-type (Fig. 5b, c; Additional file 4: Figure S4b, c). Under reducing conditions, the 48 and 50 kDa protein peaks representative of the cruciferin protomers detected under non-denaturing conditions (Additional file 4: Figure S4b, c) are almost completely eliminated and ~ 20–32.5 kDa protein peaks appear, which are likely composed of cruciferin α (27.1–31.7 kDa; Additional file 8: Table S2) and β (19.8–21.3 kDa; Additional file 8: Table S2) chains (Fig. 5b, c). While some discrepancies exist between the predicted molecular weights based on the electropherogram and molecular weights based on protein sequences (Additional file 8: Table S2), the 29 and 32.5 kDa peaks and the 20.5 and 24 kDa peaks likely represent the cruciferin α and β chains, respectively. Although the α chain peaks are not completely resolved in the electropherogram, it is evident that the highest molecular weight shoulder peak, nominally designated as 32.5 kDa (Fig. 5b), is missing in *CsCRUC*^−/−,−/−,−/−^ seeds, as per the TGX gel results, and likely represents the region where the CRUC α chain migrates. Correspondingly, a 29 kDa peak is elevated in *CsCRUC*^−/−,−/−,−/−^ seeds and is consistent with being composed of α chains from CRUA, B or D (27.1–28.6 kDa; Additional file 8: Table S2). The wild-type seed protein profile also contains a unique peak of 20.5 kDa and an elevated 24 kDa peak, likely representing the highly abundant β chains from CRUA, B, C and D (Additional file 8: Table S2). The *CsCRUC*^−/−,−/−,−/−^ seed protein profile lacks the 20.5 kDa peak and has an elevated 24 kDa peak. Although the β chains have similar molecular weights (19.9–21.3 kDa; Additional file 8: Table S2), CRUC β has several residues unique from CRUA, B and D that may confer increased negative charge and increased relative mobility during electrophoresis in the presence of SDS ([48, 49]; Additional file 5: Figure S5). This potential altered mobility combined with the approximately 50% or greater transcript abundance of *CsCRUC* vs. *CsCRUA*, *B*, and *D* (Additional file 1: Figure S1) is consistent with the 20.5 kDa peak representing CRUC β. In addition to these changes in the subunit peaks, the predicted cruciferin protomers revealed under non-denaturing conditions were also altered (Additional file 4: Figure S4b, c). The predicted CRUC protomers peak (~ 50 kDa) is decreased in *CsCRUC*^−/−,−/−,−/−^ seed protein extracts while a ~ 48 kDa peak, likely corresponding to the protomers of other cruciferins, is increased (Additional file 4: Figure S4b, c). Notably, the abundance of a 9–10 kDa peak under reducing conditions (Fig. 5b, c) and a 17.5 kDa peak under non-reducing conditions (Additional file 4: Figure S4b, c), which is likely composed of the napin large subunit and the mature napin monomer, respectively (Additional file 9: Table S3), is also increased in *CsCRUC*^−/−,−/−,−/−^ seeds. Collectively, these results demonstrate the *CsCRUC*^−/−,−/−,−/−^ line has altered abundance of seed storage proteins and that loss of CRUC is compensated by elevated levels of other seed storage proteins.

**Fig. 6:**
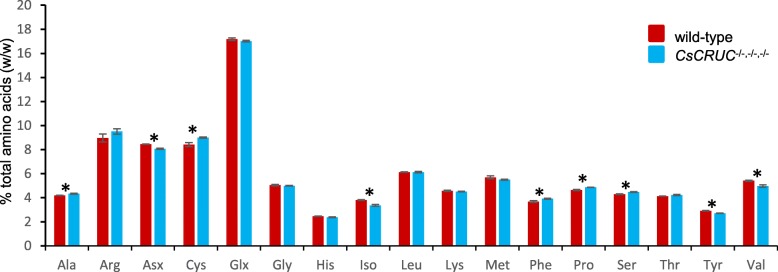
Seed amino acid composition is altered in *CsCRUC* knockout line. Means and S.E.M. for percent amino acid (w/w) are shown for triplicate measurements from each of three biological replicates. Glx represents both glutamic acid and glutamine, and Asx represents both aspartic acid and asparagine. Analysis does not include tryptophan. * indicates significant difference between wild-type and *CsCRUC*^−/−,−/−,−/−^ line (*p* < 0.05, Student’s t-test)

The redistribution of seed storage protein abundance in the *CsCRUC*^−/−,−/−,−/−^ line may also be reflected in amino acid content. The amino acid content of wild type and *CsCRUC*^−/−,−/−,−/−^ seeds was compared and a significant shift in the relative abundance of some amino acids was observed (Fig. 6). Compared to the other camelina cruciferins, CRUC has the highest content of isoleucine and tyrosine (Additional file 6: Figure S6). Correspondingly, the reduced ratio of CRUC:CRUA,B,D observed in *CsCRUC*^−/−,−/−,−/−^ seeds correlates with reduced abundance of isoleucine and tyrosine in these seeds (−11.8% and −6.9%, respectively). Similarly, CRUC has the lowest content of alanine, phenylalanine and serine amongst the cruciferins in camelina, and these amino acids are correspondingly elevated in *CsCRUC*^−/−,−/−,−/−^ seeds (+3.5%, +6.4% and +4.5%, respectively). For the remaining amino acids with significantly changed abundance, including cysteine (+7.0%), proline (+4.8%), the combined aspartate and asparagine signals (−4.5%), and valine (−8.2%), correlation with CRUC levels is less clear. Notably, CRUC has the second highest asparagine content and second lowest proline content of the camelina cruciferins, which considering the high relative level of *CsCRUC* transcription (Additional file 1: Figure S1) and protein abundance (Fig. 5) can be expected to impact levels of these amino acids in *CsCRUC*^−/−,−/−,−/−^ seeds. Additionally, amino acid levels may be affected by the elevated abundance of napins observed in *CsCRUC*^−/−,−/−,−/−^ seeds. For example, camelina napins have a seven-fold higher average cysteine content (7.0%) and 75% higher average proline content (9.4%) than in cruciferins (1.0% and 5.3%, respectively; Additional file 10: Table S4). In contrast, napins have 54% lower combined average asparagine and aspartate content (4.9%) and 33% lower average valine content (5.1%) than in cruciferins (10.7% and 7.6%, respectively). Thus, the elevated napin levels in *CsCRUC*^−/−,−/−,−/−^ seeds can be predicted to affect total amino acid levels. Collectively, the results reflect a general trend for seed amino acid levels to be influenced by the abundance of CRUC either directly through its inherent amino acid composition or indirectly by its influence on the abundance of other seed proteins.

**Fig. 7:**
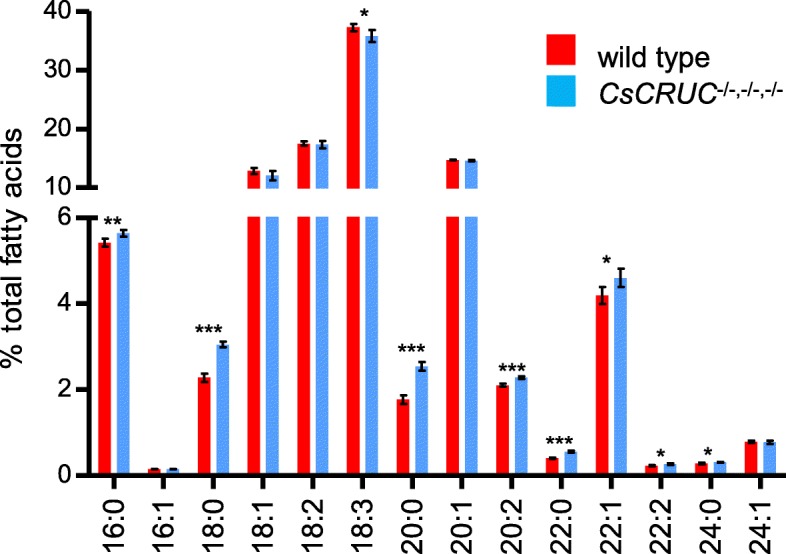
Seed fatty acid profile is altered in *CsCRUC* knockout line. Plotted values represent means ± S.D. for seed samples from five biological replicates. Fatty acids include palmitic acid (16:0), palmitoleic acid (16:1), stearic acid (18:0), oleic acid (18:1, including both delta 9 and delta 11 isomers), linoleic acid (18:2), α-linolenic acid (18:3), eicosanoic acid (20:0), eicosenoic acid (20:1), eicosadienoic acid (20:2), docosanoic acid (22:0), erucic acid (22:1), docosadienoic acid (22:2), tetracosanoic acid (24:0) and nervonic acid (24:1). * indicates significant difference between wild-type and CsCRUC^−/−,−/−,−/−^ line (*p* < 0.05, Student’s t-test)

Seed protein content is strongly related to oil content with oilseeds typically exhibiting an inverse relationship between these two storage reserves [50]. The *CsCRUC*^−/−,−/−,−/−^ seeds did not have a significant difference in seed oil content versus wild type (Table 2), in accord with the maintenance of normal seed protein content resulting from the redistributed abundance of the remaining classes of seed storage proteins. Fatty acid composition was significantly changed in *CsCRUC*^−/−,−/−,−/−^ seeds (Fig. 7; Additional file 11: Table S5). All saturated fatty acids detected were increased in relative abundance, including palmitic acid (16:0; + 4%), stearic acid (18:0; +34%), eicosanoic acid (20:0; +44%), docosanoic acid (22:0; +37%) and tetracosanoic acid (24:0; +10%). In addition, eicosadienoic acid (20:2; +8%), erucic acid (22:1; +10%), and docosadienoic acid (22:2: +12%) were increased in relative abundance. Only α-linolenic acid (18:3; −4%) was decreased in relative abundance, and all remaining detected fatty acids exhibited no significant difference from wild type. The results confirm the strong relationship between the seed content of oil and protein storage reserves, and illustrate a possible link between abundance of the different classes of seed storage proteins and seed fatty acid profile.

## Discussion

Utilisation of camelina oil in feed, biofuel or industrial feedstock applications requires increased value of the protein meal co-product to increase the underlying economic feasibility of production. As shown for other crops, this can be achieved through manipulation of seed storage protein constituents to improve desirable amino acid content either by breeding [18, 51–53] or by transgenic means to disrupt endogenous seed protein abundance [53–57]. Additionally, seed protein composition can be enhanced by transgenic approaches to express foreign proteins [53], although achieving high yields of foreign proteins in seeds requires reduction of endogenous seed storage proteins [17, 58]. In this report, we establish a camelina line that can serve as a platform for improving the value of seed meal by deploying CRISPR/Cas9 gene editing to generate a *CsCRUC* knockout line. CRUC is the most divergent at the amino acid level [37, 39] and is the most highly expressed of the cruciferin gene family [36, 38, 59], which, in combination with its unique physical attributes [39, 40], makes depletion of CRUC a prime means for investigating the potential of manipulating seed storage protein abundance for altering the nutritional value of camelina seed meal. By screening for gene editing events in four generations of a camelina lineage expressing CRISPR components, we identified a line possessing a one base pair deletion at the predicted Cas9 cut-site in the 5′ region of the first exon of *CsCRUC-G1*, *CsCRUC-G2* and *CsCRUC-G3* resulting in frame-shift leading to a premature stop codon in each gene. Seed from a line homozygous for the mutations at all three homoeologues was depleted for CRUC, confirming formation of null alleles. Seed weight and overall total protein content of the *CsCRUC* knockout seeds were not altered by depletion of CRUC protein; however, the specific protein composition of *CsCRUC* knockout seeds was altered compared to wild-type, namely the quantity of other cruciferins and napins was increased. This pattern of redistributed seed storage protein abundance without affecting total protein content was also observed in *Arabidopsis* possessing a T-DNA knockout allele of *AtCRC* [59], and in camelina and *Arabidopsis* with RNAi-mediated knockdown of napin leading to elevated cruciferin [27, 39]. These results are consistent with a proteome rebalancing model [17] wherein depletion of one class of seed storage proteins results in a compensatory effect involving elevation of other seed proteins, potentially by the redistribution of metabolic resources amongst the seed storage protein milieu. A previous attempt to knockdown cruciferin levels in camelina using RNAi was not successful [27], making the *CsCRUC* knockout reported here the first genetic tool to provide insight on the effects of cruciferins on the camelina seed proteome.

Seed meal from the *CsCRUC* knockout line had significant changes in the level of several amino acids versus wild type. This effect correlated with the altered relative abundance of the seed storage proteins in the knockout line and their respective amino acid composition. In general, amino acids that were most prevalent in CRUC were diminished in seed of the knockout line, and amino acid levels for those most prevalent in the remaining cruciferins or napins were elevated. Interestingly, no significant change in amino acid levels were observed in an *Arabidopsis* CRUC-deficient line [23], perhaps reflective of a potentially increased capacity for proteome compensation by the other seed storage proteins encoded as multi-gene families in the polyploid camelina. The extent of change in amino acid levels detected in the *CsCRUC* knockout line was consistent with the range observed by using null alleles of phaseolin in common bean (*Phaseolus vulgaris*) [52] or of conglycinin and glycinin in soybean [51], providing insight to the scope of change possible through eliminating types of seed storage proteins. Our findings demonstrate the significant impact of the balance of seed proteome constituents and its plasticity on amino acid levels, and point to opportunities to adjust seed meal nutritional value through depletion of one or more seed storage protein genes. The results also outline the potential evolutionary importance of the differential expression of seed storage proteins for camelina [38] and for the related *Arabidopsis* [36, 59] and their relative amino acid composition on establishment of an optimal storage reserve to support germination and seedling establishment.

Seeds from the *CsCRUC* knockout line did not differ in oil content from wild type, indicating the rebalanced proteome resulting from increased accumulation of other seed storage proteins maintained the relative balance of metabolic resources directed towards oil and protein synthesis during embryo development. These results contrast observations in *Arabidopsis* CRUC-deficient lines where seed oil content is reported as either being significantly increased [59] or decreased [23], and *Arabidopsis* lines deficient for combinations of the three cruciferin iosforms where oil content was decreased in all instances [39]. Interestingly, we detected distinct changes in the fatty acid profile of *CsCRUC* knockout seeds versus wild type with significant increase in the relative abundance of saturated fatty acids. Such changes were not observed in an *Arabidopsis* CRUC-deficient line [23], perhaps reflective of source-sink differences influencing metabolite availability and utilisation in the polyploid camelina. In general, the altered fatty acid profile reflects a potential subtle alteration in the flux through the fatty acid elongation pathway [60] leading to increased levels of 16:0 through to 24:0 fatty acids. Although the intracellular compartmentalisation of the elongation activities differs for the different classes of fatty acids [60], the process depends on availability of acetyl-CoA. As noted above, the *CsCRUC* knockout seeds have several changes in total amino acids levels, many of which can be catabolised to form acetyl-CoA [61] and provide a possible substrate for the fatty acid elongation pathway. Thus there may be a link between the seed proteome and oil composition or “oleaome” via sharing of metabolites initially directed towards seed storage protein synthesis. Fatty acid variants accumulate in a temporal fashion during camelina seed development [62] and coincident analysis of these in storage and membrane forms, plus seed storage proteins and free amino acids in future experiments could provide further insight to the relationship between the seed proteome and oleaome.

The polyploid nature of many crop species, including wheat, canola*,* cotton, and potato, means that gene editing-derived mutations often need to be generated and identified in multiple homoeologues for phenotypes to be exhibited [63]. Widely used methods for detecting CRISPR/Cas9 mediated mutations have a number of drawbacks in polyploids. Inexpensive methods, such as nuclease mismatch cleavage assays or restriction length polymorphism assays are not quantitative and are not sufficiently sensitive to resolve the multi-allelic nature of polyploids. In addition, sequencing of PCR-product clones or amplicons is low throughput and non-quantitative. We deployed a droplet digital PCR drop-off assay [33, 34] to detect CRISPR-derived mutations in the *CsCRUC* homoeologues and to track mutated alleles through multiple generations. This application provides a valuable method for detecting heritable gene editing events in polyploid crop species. It also provides a means to quantify the number of mutated alleles in complex plant genomes present in particular lines, which is useful when exploring gene dosage effects.

## Conclusions

The generation and analysis of a *CsCRUC* knockout line demonstrates the plasticity of the camelina seed proteome and the influence of relative amino acid content and expression levels of seed storage proteins on seed amino acid composition. Alteration of the fatty acid profile in *CsCRUC* knockout seeds reveals an intriguing link between seed protein and oil composition. The *CsCRUC* knockout line provides a platform for refining the amino acid and nutritional content of camelina seed meal by combining with other seed storage protein alleles or seed-based expression of foreign proteins. This report also establishes the ddPCR drop-off assay as a highly effective means to identify and track CRISPR-mutated alleles in polyploid genomes and gene families.

## Methods

### CRISPR/Cas9 plant transformation constructs

Plant transformation constructs for expressing CRISPR/Cas9 components were assembled by multisite Gateway cloning. An open reading frame encoding Cas9 from *Streptococcus pyogenes* with the nuclear localisation sequence (NLS) from the SV40 large T-antigen followed by the 3xFLAG epitope tag at the N-terminus and the NLS from nucleoplasmin [64] at the C-terminus, a configuration similar to that of [65], was optimised for expression in crucifers by considering codon usage of *A. thaliana* and synthesised by DNA2.0 (Menlo Park, CA, USA). The resulting *Cas9optAt* cassette (Additional file 14) was subcloned between the Gateway attL1 and attR5 sites leading to pWY454. A cassette flanked by the Gateway attL5 and attL4 sites encoding the *AtHSP18.2* (At5g59720) terminator, reported to increase expression of transgenes [66], and hygromycin-resistance cassette was assembled, resulting in pWY457. This plasmid encodes the R6Kγ origin of replication and can be propagated only in *E. coli* strains expressing the *pir* gene, a configuration enabling direct selection for recombinants from the multisite Gateway assembly reaction by selecting for hygromycin resistance after transforming a standard *E. coli* strain lacking *pir.* A gRNA expression cassette (Additional file 13) flanked by the Gateway attR4 and attL2 sites was synthesised (BioBasic, Markham, ON, Canada), termed pET28 + AtU6–26_gRNA_attR4-L2, comprising 300 bp of the promoter region of *AtU6–26* (At3g13855), including the transcription start site [41], and 92 bp of 3′ sequence intervened by a *lacZα* expression cassette flanked by asymmetric BsaI sites and linked to the gRNA scaffold sequence [67]. *CsCRUC* gRNA expression cassettes were assembled using the foregoing framework and BsaI-based Golden Gate cloning [68] to introduce a duplex oligonucleotide (Additional file 12: Table S6) encoding the desired spacer sequence, resulting in constructs pMW499 (gRNA510) and pMW501 (gRNA512). The Gateway entry clones pWY454, pWY457 and pMW499 or pMW501 were combined into the plant transformation vector pWY452 by multisite Gateway cloning using LR Clonase II (Thermo Fisher Scientific, Mississauga, ON, Canada), resulting in pMW510 and pMW512 encoding gRNA510 and gRNA512, respectively. pWY452 is a derivative of pWY109 [69] and encodes a 2.6 kb promoter fragment from *AtEF1α* (At5g60390) linked to the Gateway destination cassette followed by the CaMV 35S terminator, plus a *lacZα* expression cassette flanked by BsaI sites for Golden Gate cloning, and the *PAT* gene linked to the *EntCUP2* promoter and *NOS* terminator for selecting transgenic plants. BsaI sites in components used to develop pWY452 were eliminated using site-directed mutagenesis or resynthesised DNA segements (BioBasic) with the remaining two BsaI sites being compatible with the MoClo [70] and GoldenBraid [71] systems.

### Generation of transgenic camelina lines

*C. sativa* doubled haploid line DH55 [26] (seed provided by Isobel Parkin, Agriculture and Agri-Food Canada, with all permissions obtained) was grown in 6″ pots under growth chamber conditions (19 °C day and 15 °C night, 16 h light and 8 h dark). The apical shoot from each camelina plant was trimmed at 30–33 days post-sowing to encourage lateral bud growth. Soil was supplemented with 20–20-20 fertilizer and plants were treated for powdery mildew and insects with Senator 70WP (thiophanate-methyl; Nippon Soda) and Kontos (spirotetramat; Bayer CropScience), respectively. The constructs pMW510 and pMW512 were transformed into *Agrobacterium tumefaciens* GV3101 (pMP90) via electroporation. Plants were transformed using the floral dip method [43] with an initial treatment during the early flower bud stage and a second treatment during the full flower bud stage. Immediately after dipping, the plants were kept humid for 24 h. Seeds were harvested after approximately 100 days of growth, then sown on soil and sprayed with glufosinate-ammonium (1.5 g/L) on day 7 and day 14. Candidate T_1_ lines were validated using PCR and *Cas9optAt* (Additional file 14) specific primers (Additional file 12: Table S6). T1 lines and subsequent generations were grown under greenhouse conditions with seasonally supplemented light (16 h light, 8 h dark) when required, resulting in a generation time of approximately 3 months.

### Reverse transcription ddPCR

RNA was extracted from young leaves of T_1_ transgenic lines using the RNeasy mini kit (Qiagen) and approximately 800 ng was converted to cDNA using SuperScript™ III First-Strand Synthesis SuperMix for qRT-PCR (Invitrogen), according to instructions by the manufacturer. cDNA was diluted 10-fold and used as template in ddPCR reactions. Hydrolysis probes (generically referred to as TaqMan probes; Integrated DNA Technologies (IDT), Coralville, IA, USA) were used to assess *Cas9* expression with *CsPDF2* (Csa17g018660) as an internal standard (Additional file 12: Table S6).

### Detection of CRISPR/Cas9-derived mutations in CsCRUC by ddPCR drop-off assay

The *CsCruC* reference and indel detection hydrolysis probes and primers were designed using a combination of the PrimerQuest software (IDT) and the TaqMan Allelic Discrimination option in Primer Express 3.0 software (Applied Biosystems, Foster City, CA, USA). The drop-off probe (FAM) and reference probe (HEX) are approximately 200 base pairs apart and do not contain any SNP amongst the *CsCRUC* homoeologues. The probe and primer design also took into account sequence similarity between *CsCRUA*, *B*, *C* and *D* and considered positions of polymorphisms to ensure specificity to *CsCRUC*. All probe and primer sequences are listed in Additional file 12: Table S6.

To screen plants for CRISPR/Cas9-derived mutations in *CsCRUC* homoeologues, genomic DNA was extracted from camelina plants using the BioSprint 96 robotic workstation (Qiagen, Valencia, CA, USA) and the BioSprint 96 DNA Plant Kit (Qiagen). Approximately 500 ng of genomic DNA was digested with 5 units of EcoRI for 16 h then diluted to approximately 5 ng/μl. The 25 μl ddPCR reaction was as follows: 5 μM HEX and 5 μM FAM TaqMan probe (IDT), 18 μM forward and 18 μM reverse primer (IDT), 15–25 ng digested genomic DNA or ~ 10 pg of plasmid containing a ~ 335 base pair synthesised cassette (BioBasic, Markham, ON, Canada) of either the wild-type *CsCRUC* sequence or a four base pair deletion at the predicted Cas9 cut site for gRNA510 or gRNA512 (*CsCRUC*^*Δ4*^; Additional file 15), 12.5 μl of 2xddPCR Supermix for probes (Bio-Rad, Mississauga, ON, Canada). Droplets were generated using the QX100 Droplet Generator (Bio-Rad), transferred to a clear 96-well PCR plate (Bio-Rad) and sealed with the corresponding adhesive cover, then placed in a Model C1000 thermal cycler (Bio-Rad) with conditions as follows: 95 °C for 10 min; 35 cycles of 94 °C for 30 s and 59 °C for 30 s; 1 cycle of 95 °C for 10 min and then hold at 4 °C. Droplets were analyzed using a QX100 Droplet Reader or QX200 Droplet Reader (Bio-Rad). The two-dimensional droplet fluorescence intensity plot feature of QuantaSoft software (Bio-Rad) was used to analyse the resulting data. The droplet clusters were grouped using the QuantaSoft lasso threshold adjustment tool.

Camelina lines identified by the drop-off assay had a 480 bp fragment of *CsCruC* amplified by PCR using gene specific primers (Additional file 12: Table S6) and Taq polymerase (New England BioLabs, Whitby, ON, Canada) according to the manufacturer’s instructions. The resulting PCR product was purified using a QIAquick PCR purification kit (Qiagen), then cloned into pCR4-TOPO using a TOPO TA cloning kit (ThermoFisher). The cloned fragment from approximately 30 clones per plant was amplified by PCR and the amplicons purified using a QIAquick PCR purification kit (Qiagen) and sequenced (National Research Council, Saskatoon, SK, Canada; Eurofins Genomics, Toronto, ON, Canada).

### Seed protein extraction and protein content determination

A CryoMill (Retsch Technology, Haan, Germany) was used to grind seeds (30 seeds per sample; triplicate samples from each of three biological replicates corresponding to seed collected from individual plants of the T_6_ generation) into a fine powder in the presence of liquid nitrogen. Ground seeds were suspended in 1.5 ml seed protein extraction buffer (100 mM Tris-HCl (pH 7.5), 100 mM NaCl, 0.5 M EDTA (pH 8.0), 10 mM AEBSF, 1% (v/v) Protease Inhibitor Cocktail (Sigma P9599 supplied in DMSO as a proprietary formulation of: AEBSF, Bestatin, E-64, Leupeptin, Pepstatin A, 1,10-Phenanthroline); reduced samples included 10 mM dithiothreitol. Suspensions were centrifuged at 10,600 g in a microcentrifuge for 20 min at 4^o^ C. The supernatant was aliquoted and stored at 80 °C. Protein concentration of seed extracts was determined using a Qubit 2.0 Fluorometer (Thermo Scientific), as per the manufacturer’s instructions.

To determine protein content using nitrogen content analysis, mature camelina seeds (2–3 g) were first defatted via hexane extraction [72, 73] by being ground with ball bearings in hexane for 45 min, then filtrated to remove oil and hexanes. Defatted meal was air-dried overnight, then stored at ^−^ 20 °C. Approximately 15 mg samples of ground meal were analysed using the Flash 2000 Organic Elemental Analyzer (Thermo Scientific). A nitrogen to protein conversion factor of 6.25 was used [74]. Three samples of seeds were measured for each of three biological replicates as per above for material from the T_6_ generation. Significance of differences between wild type and CsCRUC knockout was evaluated using Student’s t-test.

### Seed protein analysis by gel and microfluidics electrophoresis

Protein extract samples (25 ng) were loaded on a TGX Stain-Free precast gel (8–16%; Bio-Rad). Seeds from three individual plants of the T_6_ generation were evaluated. To analyse seed protein composition with greater resolution the Experion Automated Electrophoresis system (Bio-Rad) and Experion Pro260 analysis kit were used. Protein samples (3 μg/μl) prepared in triplicate from seed samples from each of three individual plants of the T_6_ generation as per above were treated according to the manufacturer’s instructions. In brief, gel solution, gel stain solution, Pro260 ladder and sample buffer were prepared using Experion Pro260 analysis kit reagents; for reducing conditions dithiothreitol was included. The Experion Pro260 chip micro-channels were primed, samples were loaded and then analysed using the Experion system. The resulting electropherograms were analysed using the percentage determination function of the Experion software, which calculates each peak as a percent of the total protein species detected in a sample.

### Seed amino acid content quantification

Amino acid profiles were determined in triplicate for one defatted meal preparation from seed from each of three individual plants of the T_6_ generation [75, 76]. Defatted meal prepared for nitrogen content analysis, as described above, was weighed into 10 ml Pyrex screw cap vials with protein equivalents of 5 mg (nitrogen to protein conversion factor of 6.25). Hydrolysis was done in 2 ml of 6 M HCl (Optima grade, Fisher Scientific) with 1% (w/v) phenol for 24 h at 110 °C, with the exception of cysteine and methionine which were oxidized to cystic acid and methionine sulfone prior to 6 M HCl acid hydrolysis. Tryptophan was not assessed. DL-2 aminobutyric acid (Sigma-Aldrich) was added as the internal standard to hydrolysates after neutralization with sodium hydroxide at 0.25 mM for 6 M HCl hydrolysates and 0.2 mM for cystic acid and methionine sulfone hydrolysates. Samples were diluted five-fold with water. Neutralized hydrolysates were filtered using a 0.45 μm syringe filter and 2 ml for 6 M HCl hydrolysates or 2.5 ml for cystic acid and methionine sulfone hydrolysates applied to an Oasis HLB C18 Cartridge (Waters, Mississauga, ON, Canada), followed by an acetonitrile wash then water added for a final volume of 5 ml. Samples were stored at ^−^ 20 °C prior to pre-column derivatization using the AccQ-Fluor reagent kit (Waters). Separation and quantification of amino acids was performed using high-performance liquid chromatography (Waters Alliance 2695 HPLC, equipped with a Waters 2475 fluorescence detector with excitation wavelength of 250 nm, emission wavelength of 395 nm and gain of 15). Amino acids were resolved using multistep gradient elution with an injection volume of 5 μl. Response peaks were recorded with the Empower software (Waters). Significance of differences between wild type and *CsCRUC* knockout was evaluated using Student’s t-test.

### Seed oil content and fatty acid profile determination

Seed oil content and fatty acid composition analysis were performed as per Heydarian et al. [77] for seed samples from each of five plants for each genotype. Significance of differences between wild type and *CsCRUC* knockout was evaluated using Student’s t-test.

## Additional Files

Additional file 1: ** Figure S1.** Normalised expression values of the *CRUCIFERIN A, B, C* and *D* gene families. (PDF 157 kb)

Additional file 2: **Figure S2.** Validation of ddPCR drop-off assay to detect mutations mediated by gRNA512. (PDF 124 kb)

Additional file 3: **Figure S3.** CRISPR-derived mutations identified in *CsCRUC*. (PDF 2911 kb)

Additional file 4: **Figure S4.** Electrophoresis under non-denaturing conditions demonstrates loss of CRUC in camelina is compensated by accumulation of other seed proteins. (PDF 108 kb)

Additional file 5: **Figure S5.** CsCRUC β subunit has unique amino acids that may increase binding of SDS and increase relative mobility during electrophoresis. (PDF 112 kb)

Additional file 6: **Figure S6.** Amino acid content of cruciferin A, B, C and D in *C. sativa*. (PDF 70 kb)

Additional file 7: **Table S1.** Translated protein sequence resulting from premature stop codon in *CsCRUC* knockout alleles. (DOCX 22 kb)

Additional file 8: **Table S2.** Predicted molecular weight of camelina cruciferins and derived α and β chains. (DOCX 22 kb)

Additional file 9: **Table S3.** Predicted total molecular weight of camelina napins and derived small and large subunits. (DOCX 22 kb)

Additional file 10: **Table S4.** Amino acid content of camelina cruciferins and napins. (DOCX 23 kb)

Additional file 11: **Table S5.** Fatty acid profile of *CsCRUC* knockout seeds. (DOCX 13 kb)

Additional file 12: **Table S6.** Primer, probe and oligonucleotide sequences. (DOCX 23 kb)

Additional file 13: DNA sequence of gRNA expression cassette. (DOCX 25 kb)

Additional file 14: DNA sequence of *Cas9optAt* optimised for general expression in crucifers. (DOCX 27 kb)

Additional file 15: DNA sequences of synthesised cassettes used to validate drop-off assays. (DOCX 25 kb)

## Data Availability

All results and data for this investigation are presented in the enclosed figures and additional files. Materials developd in this study may be obtained from the corresponding author, subject to policies of the author’s institution.

## Abbreviations

CRISPR: Clustered Regularly Interspaced Short Palindromic Repeats
CRU: CRUCIFERIN
ddPCR: droplet digital polymerase chain reaction
