# Supplementary material for: CRISPR/Cas9 editing of three CRUCIFERIN C homoeologues alters the seed protein profile in Camelina sativa
Source: BMC Plant Biol. 2019 Jul 4;19:292. doi: 10.1186/s12870-019-1873-0 (PMC6611024; doi:10.1186/s12870-019-1873-0)
Supplement: Supplementary file 1 — Figure S1. Normalised expression values of the CRUCIFERIN A, B, C and D gene families. (PDF 157 kb) [file 12870_2019_1873_MOESM1_ESM.pdf]

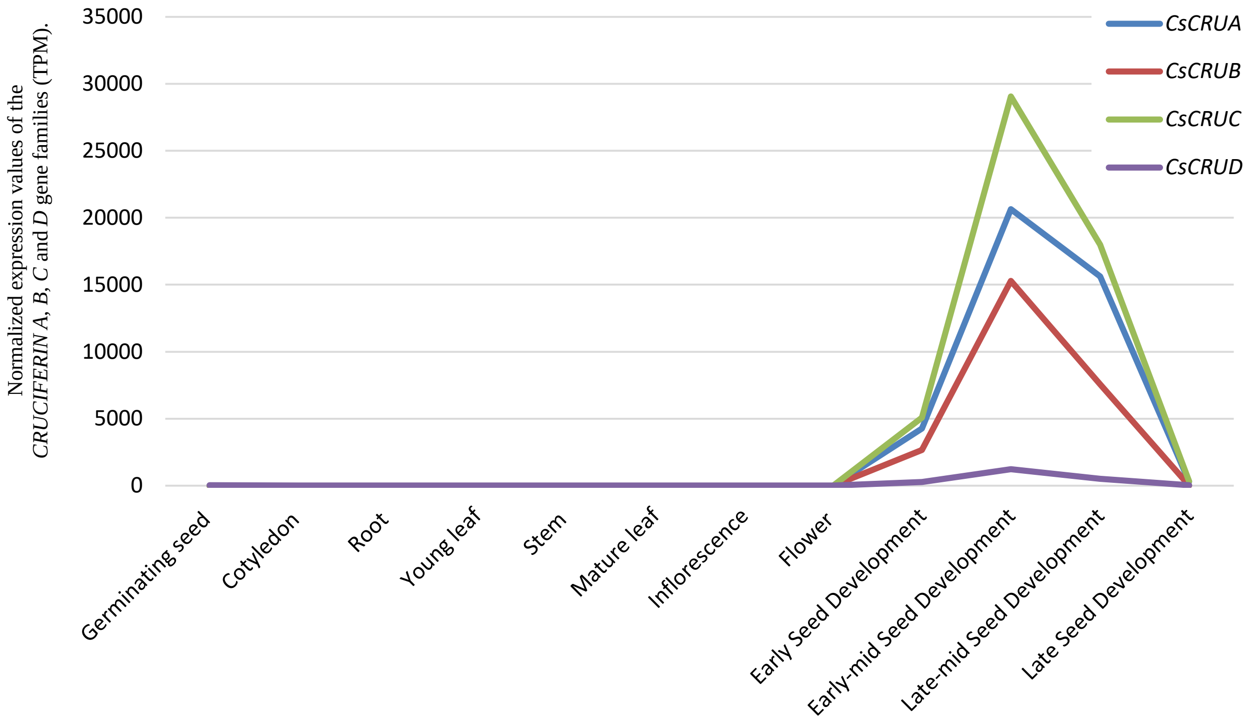

Figure S1. Normalised expression values of the *CRUCIFERIN A, B, C* and *D* gene families. Values (transcripts per kilobase million (TPM)) are the sum of the three homoeologues from each family. Data was taken from [38] http://bar.utoronto.ca/efp_camelina/cgi-bin/efpWeb.cgi.
