# Supplementary material for: CRISPR/Cas9 editing of three CRUCIFERIN C homoeologues alters the seed protein profile in Camelina sativa
Source: BMC Plant Biol. 2019 Jul 4;19:292. doi: 10.1186/s12870-019-1873-0 (PMC6611024; doi:10.1186/s12870-019-1873-0)
Supplement: Supplementary file 2 — Figure S2. Validation of ddPCR drop-off assay to detect mutations mediated by gRNA512. (PDF 124 kb) [file 12870_2019_1873_MOESM2_ESM.pdf]

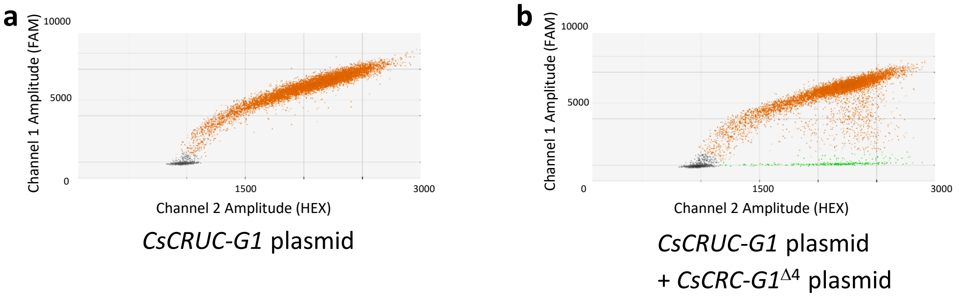

Figure S2. Validation of ddPCR drop-off assay to detect mutations mediated by gRNA512.

a *CsCRUC-G1* plasmid generates amplicons in which both drop-off (FAM) probe and reference probe (HEX) bind, resulting in only double-positive droplets. **b** A mixture *CsCRUC-G1* plasmid and *CsCRUC-G1*$^{\Delta 4}$ plasmid generate wild-type amplicons in which both drop-off (FAM) probe and reference probe (HEX) bind, resulting in double-positive droplets, and Δ4 mutation-containing amplicons in which only the reference probe (HEX) binds, resulting in single-positive droplets.
