# Supplementary material for: CRISPR/Cas9 editing of three CRUCIFERIN C homoeologues alters the seed protein profile in Camelina sativa
Source: BMC Plant Biol. 2019 Jul 4;19:292. doi: 10.1186/s12870-019-1873-0 (PMC6611024; doi:10.1186/s12870-019-1873-0)
Supplement: Supplementary file 3 — Figure S3. CRISPR-derived mutations identified in CsCRUC. (PDF 2911 kb) [file 12870_2019_1873_MOESM3_ESM.pdf]

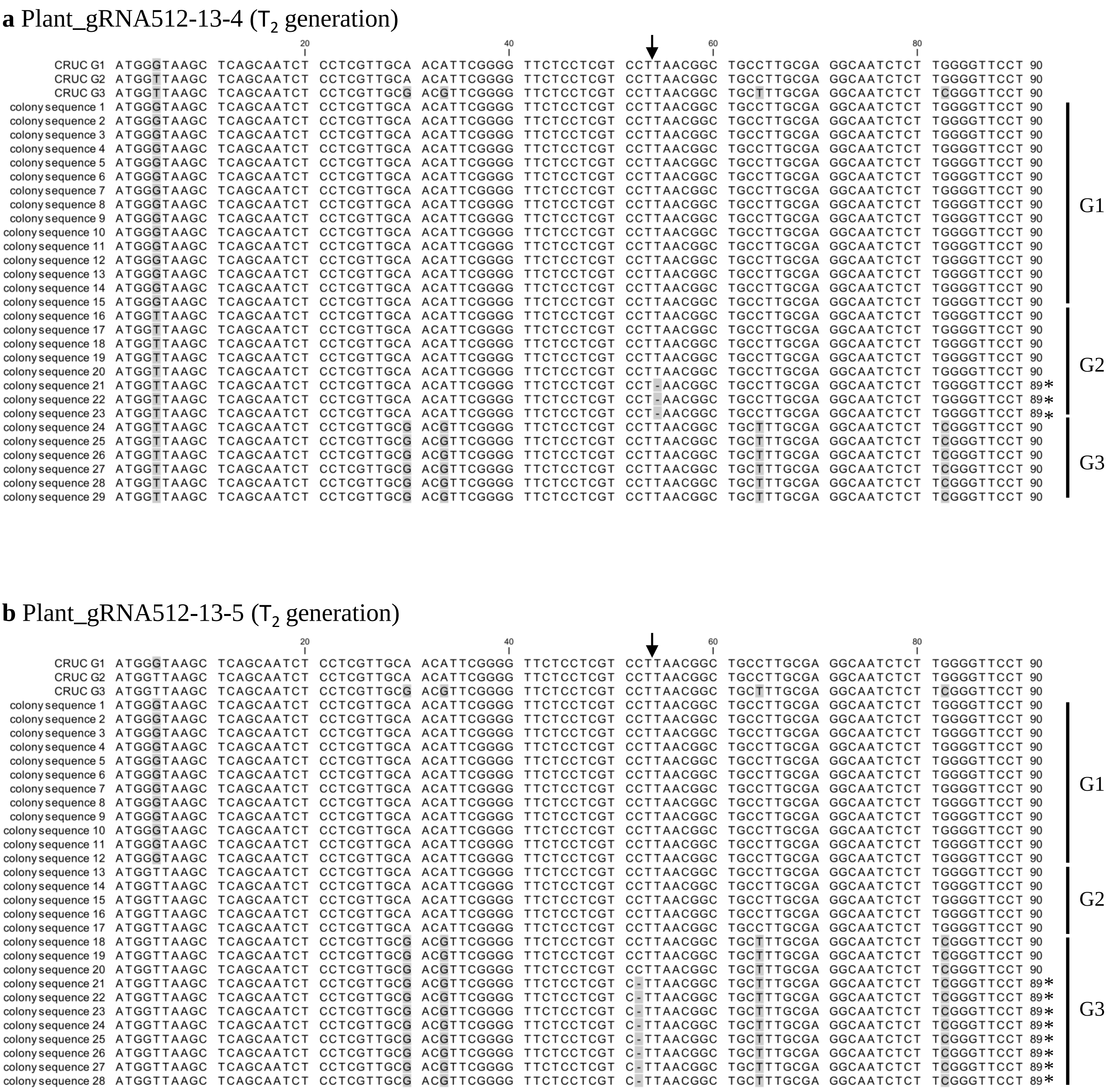
a Plant_gRNA512-13-4 (T_2 generation)
20
40
60
80
CRUC G1 ATGGGTAAGC TCAGCAATCT CCTCGTTGCA ACATTCGGGG TTCTCCTCGT CCTTAACGGC TGCCTTGCGA GGCAATCTCT TGGGGTTCCT 90
CRUC G2 ATGGTTAAGC TCAGCAATCT CCTCGTTGCA ACATTCGGGG TTCTCCTCGT CCTTAACGGC TGCCTTGCGA GGCAATCTCT TGGGGTTCCT 90
CRUC G3 ATGGTTAAGC TCAGCAATCT CCTCGTTGCG ACGTTCGGGG TTCTCCTCGT CCTTAACGGC TGCTTTGCGA GGCAATCTCT TCGGGTTCCT 90
colony sequence 21 ATGGTTAAGC TCAGCAATCT CCTCGTTGCA ACATTCGGGG TTCTCCTCGT CCT-AACGGC TGCCTTGCGA GGCAATCTCT TGGGGTTCCT 89*
G1
G2
G3
b Plant_gRNA512-13-5 (T_2 generation)
colony sequence 21 ATGGTTAAGC TCAGCAATCT CCTCGTTGCG ACGTTCGGGG TTCTCCTCGT C-TTAACGGC TGCTTTGCGA GGCAATCTCT TCGGGTTCCT 89*
G1
G2
G3

c Plant_gRNA512-13-6 (T_2 generation)

```
                                                     20                            40                 ↓          60                            80
CRUC G1              ATGGGTAAGC TCAGCAATCT CCTCGTTGCA ACATTCGGGG TTCTCCTCGT CCTTAACGGC TGCCTTGCGA GGCAATCTCT TGGGGTTCCT 90
CRUC G2              ATGGTTAAGC TCAGCAATCT CCTCGTTGCA ACATTCGGGG TTCTCCTCGT CCTTAACGGC TGCCTTGCGA GGCAATCTCT TGGGGTTCCT 90
CRUC G3              ATGGTTAAGC TCAGCAATCT CCTCGTTGCG ACGTTCGGGG TTCTCCTCGT CCTTAACGGC TGCTTTGCGA GGCAATCTCT TCGGGTTCCT 90
colony sequence 1    ATGGGTAAGC TCAGCAATCT CCTCGTTGCA ACATTCGGGG TTCTCCTCGT CCTTAACGGC TGCCTTGCGA GGCAATCTCT TGGGGTTCCT 90    G1
colony sequence 2    ATGGGTAAGC TCAGCAATCT CCTCGTTGCA ACATTCGGGG TTCTCCTCGT CCTTAACGGC TGCCTTGCGA GGCAATCTCT TGGGGTTCCT 90    G1
colony sequence 3    ATGGGTAAGC TCAGCAATCT CCTCGTTGCA ACATTCGGGG TTCTCCTCGT CCTTAACGGC TGCCTTGCGA GGCAATCTCT TGGGGTTCCT 90    G1
colony sequence 4    ATGGGTAAGC TCAGCAATCT CCTCGTTGCA ACATTCGGGG TTCTCCTCGT CCTTAACGGC TGCCTTGCGA GGCAATCTCT TGGGGTTCCT 90    G1
colony sequence 5    ATGGGTAAGC TCAGCAATCT CCTCGTTGCA ACATTCGGGG TTCTCCTCGT CCTTAACGGC TGCCTTGCGA GGCAATCTCT TGGGGTTCCT 90    G1
colony sequence 6    ATGGGTAAGC TCAGCAATCT CCTCGTTGCA ACATTCGGGG TTCTCCTCGT CCT-AACGGC TGCCTTGCGA GGCAATCTCT TGGGGTTCCT 89 *  G1
colony sequence 7    ATGGGTAAGC TCAGCAATCT CCTCGTTGCA ACATTCGGGG TTCTCCTCGT CCT-AACGGC TGCCTTGCGA GGCAATCTCT TGGGGTTCCT 89 *  G1
colony sequence 8    ATGGGTAAGC TCAGCAATCT CCTCGTTGCA ACATTCGGGG TTCTCCTCGT CCT-AACGGC TGCCTTGCGA GGCAATCTCT TGGGGTTCCT 89 *  G1
colony sequence 9    ATGGGTAAGC TCAGCAATCT CCTCGTTGCA ACATTCGGGG TTCTCCTCGT CCT-AACGGC TGCCTTGCGA GGCAATCTCT TGGGGTTCCT 89 *  G1
colony sequence 10   ATGGGTAAGC TCAGCAATCT CCTCGTTGCA ACATTCGGGG TTCTCCTCGT CCT-AACGGC TGCCTTGCGA GGCAATCTCT TGGGGTTCCT 89 *  G1
colony sequence 11   ATGGGTAAGC TCAGCAATCT CCTCGTTGCA ACATTCGGGG TTCTCCTCGT CCT-AACGGC TGCCTTGCGA GGCAATCTCT TGGGGTTCCT 89 *  G1
colony sequence 12   ATGGGTAAGC TCAGCAATCT CCTCGTTGCA ACATTCGGGG TTCTCCTCGT CCT-AACGGC TGCCTTGCGA GGCAATCTCT TGGGGTTCCT 89 *  G1
colony sequence 13   ATGGTTAAGC TCAGCAATCT CCTCGTTGCA ACATTCGGGG TTCTCCTCGT CCTTAACGGC TGCCTTGCGA GGCAATCTCT TGGGGTTCCT 90    G2
colony sequence 14   ATGGTTAAGC TCAGCAATCT CCTCGTTGCA ACATTCGGGG TTCTCCTCGT CCTTAACGGC TGCCTTGCGA GGCAATCTCT TGGGGTTCCT 90    G2
colony sequence 15   ATGGTTAAGC TCAGCAATCT CCTCGTTGCA ACATTCGGGG TTCTCCTCGT CCTTAACGGC TGCCTTGCGA GGCAATCTCT TGGGGTTCCT 90    G2
colony sequence 16   ATGGTTAAGC TCAGCAATCT CCTCGTTGCA ACATTCGGGG TTCTCCTCGT CCTTAACGGC TGCCTTGCGA GGCAATCTCT TGGGGTTCCT 90    G2
colony sequence 17   ATGGTTAAGC TCAGCAATCT CCTCGTTGCA ACATTCGGGG TTCTCCTCGT CCTTAACGGC TGCCTTGCGA GGCAATCTCT TGGGGTTCCT 90    G2
colony sequence 18   ATGGTTAAGC TCAGCAATCT CCTCGTTGCA ACATTCGGGG TTCTCCTCGT CCTTAACGGC TGCCTTGCGA GGCAATCTCT TGGGGTTCCT 90    G2
colony sequence 19   ATGGTTAAGC TCAGCAATCT CCTCGTTGCA ACATTCGGGG TTCTCCTCGT CCTTAACGGC TGCCTTGCGA GGCAATCTCT TGGGGTTCCT 90    G2
colony sequence 20   ATGGTTAAGC TCAGCAATCT CCTCGTTGCA ACATTCGGGG TTCTCCTCGT CCTTAACGGC TGCCTTGCGA GGCAATCTCT TGGGGTTCCT 90    G2
colony sequence 21   ATGGTTAAGC TCAGCAATCT CCTCGTTGCA ACATTCGGGG TTCTCCTCGT CCTTAACGGC TGCCTTGCGA GGCAATCTCT TGGGGTTCCT 90    G2
colony sequence 22   ATGGTTAAGC TCAGCAATCT CCTCGTTGCA ACATTCGGGG TTCTCCTCGT CCTTAACGGC TGCCTTGCGA GGCAATCTCT TGGGGTTCCT 90    G2
colony sequence 23   ATGGTTAAGC TCAGCAATCT CCTCGTTGCG ACGTTCGGGG TTCTCCTCGT CCT-AACGGC TGCTTTGCGA GGCAATCTCT TCGGGTTCCT 89 Ŧ  G3
colony sequence 24   ATGGTTAAGC TCAGCAATCT CCTCGTTGCG ACGTTCGGGG TTCTCCTCGT CCTTAACGGC TGCTTTGCGA GGCAATCTCT TCGGGTTCCT 90    G3
colony sequence 25   ATGGTTAAGC TCAGCAATCT CCTCGTTGCG ACGTTCGGGG TTCTCCTCGT CCTTAACGGC TGCTTTGCGA GGCAATCTCT TCGGGTTCCT 90    G3
colony sequence 26   ATGGTTAAGC TCAGCAATCT CCTCGTTGCG ACGTTCGGGG TTCTCCTCGT CCTTAACGGC TGCTTTGCGA GGCAATCTCT TCGGGTTCCT 90    G3
colony sequence 27   ATGGTTAAGC TCAGCAATCT CCTCGTTGCG ACGTTCGGGG TTCTCCTCGT CCTTAACGGC TGCTTTGCGA GGCAATCTCT TCGGGTTCCT 90    G3
colony sequence 28   ATGGTTAAGC TCAGCAATCT CCTCGTTGCG ACGTTCGGGG TTCTCCTCGT CCTTAACGGC TGCTTTGCGA GGCAATCTCT TCGGGTTCCT 90    G3
colony sequence 29   ATGGTTAAGC TCAGCAATCT CCTCGTTGCG ACGTTCGGGG TTCTCCTCGT CCTTAACGGC TGCTTTGCGA GGCAATCTCT TCGGGTTCCT 90    G3
colony seqeunce 30   ATGGTTAAGC TCAGCAATCT CCTCGTTGCG ACGTTCGGGG TTCTCCTCGT CCTTAACGGC TGCTTTGCGA GGCAATCTCT TCGGGTTCCT 90    G3
colony sequence 31   ATGGTTAAGC TCAGCAATCT CCTCGTTGCG ACGTTCGGGG TTCTCCTCGT CCTTAACGGC TGCTTTGCGA GGCAATCTCT TCGGGTTCCT 90    G3
```

d Plant_gRNA512-13-7(T_2 generation)

```
                                                     20                            40                 ↓          60                            80
CRUC G1              ATGGGTAAGC TCAGCAATCT CCTCGTTGCA ACATTCGGGG TTCTCCTCGT CCTT-AACGG CTGCCTTGCG AGGCAATCTC TTGGGGTTCC 89
CRUC G2              ATGGTTAAGC TCAGCAATCT CCTCGTTGCA ACATTCGGGG TTCTCCTCGT CCTT-AACGG CTGCCTTGCG AGGCAATCTC TTGGGGTTCC 89
CRUC G3              ATGGTTAAGC TCAGCAATCT CCTCGTTGCG ACGTTCGGGG TTCTCCTCGT CCTT-AACGG CTGCTTTGCG AGGCAATCTC TTCGGGTTCC 89
colony sequence 1    ATGGGTAAGC TCAGCAATCT CCTCGTTGCA ACATTCGGGG TTCTCCTCGT CCTT-AACGG CTGCCTTGCG AGGCAATCTC TTGGGGTTCC 89    G1
colony sequence 2    ATGGGTAAGC TCAGCAATCT CCTCGTTGCA ACATTCGGGG TTCTCCTCGT CCTT-AACGG CTGCCTTGCG AGGCAATCTC TTGGGGTTCC 89    G1
colony sequence 3    ATGGGTAAGC TCAGCAATCT CCTCGTTGCA ACATTCGGGG TTCTCCTCGT CCTT-AACGG CTGCCTTGCG AGGCAATCTC TTGGGGTTCC 89    G1
colony sequence 4    ATGGTTAAGC TCAGCAATCT CCTCGTTGCA ACATTCGGGG TTCTCCTCGT CCTT-AACGG CTGCCTTGCG AGGCAATCTC TTGGGGTTCC 89    G1
colony sequence 5    ATGGGTAAGC TCAGCAATCT CCTCGTTGCA ACATTCGGGG TTCTCCTCGT CCTT-AACGG CTGCCTTGCG AGGCAATCTC TTGGGGTTCC 89    G1
colony sequence 6    ATGGGTAAGC TCAGCAATCT CCTCGTTGCA ACATTCGGGG TTCTCCTCGT CCTT-AACGG CTGCCTTGCG AGGCAATCTC TTGGGGTTCC 89    G1
colony sequence 7    ATGGGTAAGC TCAGCAATCT CCTCGTTGCA ACATTCGGGG TTCTCCTCGT CCTT-AACGG CTGCCTTGCG AGGCAATCTC TTGGGGTTCC 89    G1
colony sequence 8    ATGGGTAAGC TCAGCAATCT CCTCGTTGCA ACATTCGGGG TTCTCCTCGT CCTTTAACGG CTGCCTTGCG AGGCAATCTC TTGGGGTTCC 90 *  G1
colony sequence 9    ATGGGTAAGC TCAGCAATCT CCTCGTTGCA ACATTCGGGG TTCTCCTCGT CCTTTAACGG CTGCCTTGCG AGGCAATCTC TTGGGGTTCC 90 *  G1
colony sequence 10   ATGGGTAAGC TCAGCAATCT CCTCGTTGCA ACATTCGGGG TTCTCCTCGT CCTTTAACGG CTGCCTTGCG AGGCAATCTC TTGGGGTTCC 90 *  G1
colony sequence 11   ATGGGTAAGC TCAGCAATCT CCTCGTTGCA ACATTCGGGG TTCTCCTCGT CCTTTAACGG CTGCCTTGCG AGGCAATCTC TTGGGGTTCC 90 *  G1
colony sequence 12   ATGGGTAAGC TCAGCAATCT CCTCGTTGCA ACATTCGGGG TTCTCCTCGT CCTTTAACGG CTGCCTTGCG AGGCAATCTC TTGGGGTTCC 90 *  G1
colony sequence 13   ATGGGTAAGC TCAGCAATCT CCTCGTTGCA ACATTCGGGG TTCTCCTCGT CCTTTAACGG CTGCCTTGCG AGGCAATCTC TTGGGGTTCC 90 *  G1
colony seqeunce 14   ATGGGTAAGC TCAGCAATCT CCTCGTTGCA ACATTCGGGG TTCTCCTCGT CCTTTAACGG CTGCCTTGCG AGGCAATCTC TTGGGGTTCC 90 *  G1
colony sequence 15   ATGGGTAAGC TCAGCAATCT CCTCGTTGCA ACATTCGGGG TTCTCCTCGT CCT--AACGG CTGCCTTGCG AGGCAATCTC TTGGGGTTCC 88 Ŧ  G1
colony sequence 16   ATGGTTAAGC TCAGCAATCT CCTCGTTGCA ACATTCGGGG TTCTCCTCGT CCT--AACGG CTGCCTTGCG AGGCAATCTC TTGGGGTTCC 88 *  G2
colony sequence 17   ATGGTTAAGC TCAGCAATCT CCTCGTTGCA ACATTCGGGG TTCTCCTCGT CCT--AACGG CTGCCTTGCG AGGCAATCTC TTGGGGTTCC 88 *  G2
colony sequence 18   ATGGTTAAGC TCAGCAATCT CCTCGTTGCA ACATTCGGGG TTCTCCTCGT CCT--AACGG CTGCCTTGCG AGGCAATCTC TTGGGGTTCC 88 *  G2
colony sequence 19   ATGGTTAAGC TCAGCAATCT CCTCGTTGCA ACATTCGGGG TTCTCCTCGT CCT--AACGG CTGCCTTGCG AGGCAATCTC TTGGGGTTCC 88 *  G2
colony sequence 20   ATGGTTAAGC TCAGCAATCT CCTCGTTGCA ACATTCGGGG TTCTCCTCGT CCT--AACGG CTGCCTTGCG AGGCAATCTC TTGGGGTTCC 88 *  G2
colony sequence 21   ATGGTTAAGC TCAGCAATCT CCTCGTTGCA ACATTCGGGG TTCTCCTCGT CCT--AACGG CTGCCTTGCG AGGCAATCTC TTGGGGTTCC 88 *  G2
colony sequence 22   ATGGTTAAGC TCAGCAATCT CCTCGTTGCA ACATTCGGGG TTCTCCTCGT CCTT-AACGG CTGCCTTGCG AGGCAATCTC TTGGGGTTCC 89    G2
colony sequence 23   ATGGTTAAGC TCAGCAATCT CCTCGCTGCA ACATTCGGGG TTCTCCTCGT CCTT-AACGG CTGCCTTGCG AGGCAATCTC TTGGGGTTCC 89    G2
colony sequence 24   ATGGTTAAGC TCAGCAATCT CCTCGTTGCG ACGTTCGGGG TTCTCCTCGT CCTT-AACGG CTGCTTTGCG AGGCAATCTC TTCGGGTTCC 89    G3
colony sequence 25   ATGGTTAAGC TCAGCAATCT CCTCGTTGCG ACGTTCGGGG TTCTCCTCGT CCTT-AACGG CTGCTTTGCG AGGCAATCTC TTCGGGTTCC 89    G3
colony sequence 26   ATGGTTAAGC TCAGCAATCT CCTCGTTGCG ACGTTCGGGG TTCTCCTCGT CCTT-AACGG CTGCTTTGCG AGGCAATCTC TTCGGGTTCC 89    G3
```

e Plant_gRNA512-13-10 (T_2 generation)

		20		40		60		80				
CRUC G1	ATGGGTAAGC	TCAGCAATCT	CCTCGTTGCA	ACATTCGGGG	TTCTCCTCGT	CCTTAACGGC	TGCCTTGCGA	GGCAATCTCT	TGGGGTTCCT	90		
CRUC G2	ATGGTTAAGC	TCAGCAATCT	CCTCGTTGCA	ACATTCGGGG	TTCTCCTCGT	CCTTAACGGC	TGCCTTGCGA	GGCAATCTCT	TGGGGTTCCT	90		
CRUC G3	ATGGTTAAGC	TCAGCAATCT	CCTCGTTGCG	ACGTTCGGGG	TTCTCCTCGT	CCTTAACGGC	TGCTTTGCGA	GGCAATCTCT	TCGGGTTCCT	90		
colony sequence 1	ATGGGTAAGC	TCAGCAATCT	CCTCGTTGCA	ACATTCGGGG	TTCTCCTCGT	CCTTAACGGC	TGCCTTGCGA	GGCAATCTCT	TGGGGTTCCT	90		
colony sequence 2	ATGGGTAAGC	TCAGCAATCT	CCTCGTTGCA	ACATTCGGGG	TTCTCCTCGT	CCTTAACGGC	TGCCTTGCGA	GGCAATCTCT	TGGGGTTCCT	90		
colony sequence 3	ATGGGTAAGC	TCAGCAATCT	CCTCGTTGCA	ACATTCGGGG	TTCTCCTCGT	CCTTAACGGC	TGCCTTGCGA	GGCAATCTCT	TGGGGTTCCT	90		
colony sequence 4	ATGGGTAAGC	TCAGCAATCT	CCTCGTTGCA	ACATTCGGGG	TTCTCCTCGT	---TAACGGC	TGCCTTGCGA	GGCAATCTCT	TGGGGTTCCT	87	*	
colony sequence 5	ATGGGTAAGC	TCAGCAATCT	CCTCGTTGCA	ACATTCGGGG	TTCTCCTCGT	---TAACGGC	TGCCTTGCGA	GGCAATCTCT	TGGGGTTCCT	87	*	
colony sequence 6	ATGGGTAAGC	TCAGCAATCT	CCTCGTTGCA	ACATTCGGGG	TTCTCCTCGT	---TAACGGC	TGCCTTGCGA	GGCAATCTCT	TGGGGTTCCT	87	*	
colony sequence 7	ATGGGTAAGC	TCAGCAATCT	CCTCGTTGCA	ACATTCGGGG	TTCTCCTCGT	---TAACGGC	TGCCTTGCGA	GGCAATCTCT	TGGGGTTCCT	87	*	
colony sequence 8	ATGGGTAAGC	TCAGCAATCT	CCTCGTTGCA	ACATTCGGGG	TTCTCCTCGT	---TAACGGC	TGCCTTGCGA	GGCAATCTCT	TGGGGTTCCT	87	*	
colony sequence 9	ATGGGTAAGC	TCAGCAATCT	CCTCGTTGCA	ACATTCGGGG	TTCTCCTCGT	---TAACGGC	TGCCTTGCGA	GGCAATCTCT	TGGGGTTCCT	87	*	G1
colony sequence 10	ATGGGTAAGC	TCAGCAATCT	CCTCGTTGCA	ACATTCGGGG	TTCTCCTCGT	---TAACGGC	TGCCTTGCGA	GGCAATCTCT	TGGGGTTCCT	87	*	
colony sequence 11	ATGGGTAAGC	TCAGCAATCT	CCTCGTTGCA	ACATTCGGGG	TTCTCCTCGT	---TAACGGC	TGCCTTGCGA	GGCAATCTCT	TGGGGTTCCT	87	*	
colony sequence 12	ATGGGTAAGC	TCAGCAATCT	CCTCGTTGCA	ACATTCGGGG	TTCTCCTCGT	---TAACGGC	TGCCTTGCGA	GGCAATCTCT	TGGGGTTCCT	87	*	
colony sequence 13	ATGGGTAAGC	TCAGCAATCT	CCTCGTTGCA	ACATTCGGGG	TTCTCCTCGT	CCTTAACGGC	TGCCTTGCGA	GGCAATCTCT	TGGGGTTCCT	90		
colony sequence 14	ATGGGTAAGC	TCAGCAATCT	CCTCGTTGCA	ACATTCGGGG	TTCTCCTCGT	CCTTAACGGC	TGCCTTGCGA	GGCAATCTCT	TGGGGTTCCT	90		
colony sequence 15	ATGGTTAAGC	TCAGCAATCT	CCTCGTTGCA	ACATTCGGGG	TTCTCCTCGT	CCTTAACGGC	TGCCTTGCGA	GGCAATCTCT	TGGGGTTCCT	90		
colony sequence 16	ATGGTTAAGC	TCAGCAATCT	CCTCGTTGCA	ACATTCGGGG	TTCTCCTCGT	CCTTAACGGC	TGCCTTGCGA	GGCAATCTCT	TGGGGTTCCT	90		
colony sequence 17	ATGGTTAAGC	TCAGCAATCT	CCTCGTTGCA	ACATTCGGGG	TTCTCCTCGT	CCTTAACGGC	TGCCTTGCGA	GGCAATCTCT	TGGGGTTCCT	90		
colony sequence 18	ATGGTTAAGC	TCAGCAATCT	CCTCGTTGCA	ACATTCGGGG	TTCTCCTCGT	CCTTAACGGC	TGCCTTGCGA	GGCAATCTCT	TGGGGTTCCT	90		
colony sequence 19	ATGGTTAAGC	TCAGCAATCT	CCTCGTTGCA	ACATTCGGGG	TTCTCCTCGT	CCTTAACGGC	TGCCTTGCGA	GGCAATCTCT	TGGGGTTCCT	90		G2
colony sequence 20	ATGGTTAAGC	TCAGCAATCT	CCTCGTTGCA	ACATTCGGGG	TTCTCCTCGT	CCTTAACGGC	TGCCTTGCGA	GGCAATCTCT	TGGGGTTCCT	90		
colony sequence 21	ATGGTTAAGC	TCAGCAATCT	CCTCGTTGCA	ACATTCGGGG	TTCTCCTCGT	CCTTAACGGC	TGCCTTGCGA	GGCAATCTCT	TGGGGTTCCT	90		
colony sequence 22	ATGGTTAAGC	TCAGCAATCT	CCTCGTTGCA	ACATTCGGGG	TTCTCCTCGT	CCTTAACGGC	TGCCTTGCGA	GGCAATCTCT	TGGGGTTCCT	90		
colony sequence 23	ATGGTTAAGC	TCAGCAATCT	CCTCGTTGCG	ACGTTCGGGG	TTCTCCTCGT	CCTTAACGGC	TGCTTTGCGA	GGCAATCTCT	TCGGGTTCCT	90		
colony sequence 24	ATGGTTAAGC	TCAGCAATCT	CCTCGTTGCG	ACGTTCGGGG	TTCTCCTCGT	CCTTAACGGC	TGCTTTGCGA	GGCAATCTCT	TCGGGTTCCT	90		
colony sequence 25	ATGGTTAAGC	TCAGCAATCT	CCTCGTTGCG	ACGTTCGGGG	TTCTCCTCGT	CCTTAACGGC	TGCTTTGCGA	GGCAATCTCT	TCGGGTTCCT	90		
colony sequence 26	ATGGTTAAGC	TCAGCAATCT	CCTCGTTGCG	ACGTTCGGGG	TTCTCCTCGT	CCTTAACGGC	TGCTTTGCGA	GGCAATCTCT	TCGGGTTCCT	90		G3
colony sequence 27	ATGGTTAAGC	TCAGCAATCT	CCTCGTTGCG	ACGTTCGGGG	TTCTCCTCGT	CCTTAACGGC	TGCTTTGCGA	GGCAATCTCT	TCGGGTTCCT	90		
colony sequence 28	ATGGTTAAGC	TCAGCAATCT	CCTCGTTGCG	ACGTTCGGGG	TTCTCCTCGT	CCTTAACGGC	TGCTTTGCGA	GGCAATCTCT	TCGGGTTCCT	90		
colony sequence 29	ATGGTTAAGC	TCAGCAATCT	CCTCGTTGCG	ACGTTCGGGG	TTCTCCTCGT	CCTTAACGGC	TGCTTTGCGA	GGCAATCTCT	TCGGGTTCCT	90		

f Plant_gRNA512-1-46 (T_2 generation)

		20		40		60		80				
CRUC G1	ATGGGTAAGC	TCAGCAATCT	CCTCGTTGCA	ACATTCGGGG	TTCTCCTCGT	CCT-TAACGG	CTGCCTTGCG	AGGCAATCTC	TTGGGGTTCC	89		
CRUC G2	ATGGTTAAGC	TCAGCAATCT	CCTCGTTGCA	ACATTCGGGG	TTCTCCTCGT	CCT-TAACGG	CTGCCTTGCG	AGGCAATCTC	TTGGGGTTCC	89		
CRUC G3	ATGGTTAAGC	TCAGCAATCT	CCTCGTTGCG	ACGTTCGGGG	TTCTCCTCGT	CCT-TAACGG	CTGCTTTGCG	AGGCAATCTC	TTCGGGTTCC	89		
colony sequence 1	ATGGGTAAGC	TCAGCAATCT	CCTCGTTGCA	ACATTCGGGG	TTCTCCTCGT	CCT-TAACGG	CTGCCTTGCG	AGGCAATCTC	TTGGGGTTCC	89		
colony sequence 2	ATGGGTAAGC	TCAGCAATCT	CCTCGTTGCA	ACATTCGGGG	TTCTCCTCGT	CCT-TAACGG	CTGCCTTGCG	AGGCAATCTC	TTGGGGTTCC	89		
colony seqeunce 3	ATGGGTAAGC	TCAGCAATCT	CCTCGTTGCA	ACATTCGGGG	TTCTCCTCGT	CCT-TAACGG	CTGCCTTGCG	AGGCAATCTC	TTGGGGTTCC	89		
colony sequence 4	ATGGGTAAGC	TCAGCAATCT	CCTCGTTGCA	ACATTCGGGG	TTCTCCTCGT	CCT-TAACGG	CTGCCTTGCG	AGGCAATCTC	TTGGGGTTCC	89		
colony sequence 5	ATGGGTAAGC	TCAGCAATCT	CCTCGTTGCA	ACATTCGGGG	TTCTCCTCGT	CCTATAACGG	CTGCCTTGCG	AGGCAATCTC	TTGGGGTTCC	90	*	
colony sequence 6	ATGGGTAAGC	TCAGCAATCT	CCTCGTTGCA	ACATTCGGGG	TTCTCCTCGT	CCTATAACGG	CTGCCTTGCG	AGGCAATCTC	TTGGGGTTCC	90	*	
colony sequence 7	ATGGGTAAGC	TCAGCAATCT	CCTCGTTGCA	ACATTCGGGG	TTCTCCTCGT	CCTATAACGG	CTGCCTTGCG	AGGCAATCTC	TTGGGGTTCC	90	*	G1
colony sequence 8	ATGGGTAAGC	TCAGCAATCT	CCTCGTTGCA	ACATTCGGGG	TTCTCCTCGT	CCTATAACGG	CTGCCTTGCG	AGGCAATCTC	TTGGGGTTCC	90	*	
colony sequence 9	ATGGGTAAGC	TCAGCAATCT	CCTCGTTGCA	ACATTCGGGG	TTCTCCTCGT	CCTATAACGG	CTGCCTTGCG	AGGCAATCTC	TTGGGGTTCC	90	*	
colony sequence 10	ATGGGTAAGC	TCAGCAATCT	CCTCGTTGCA	ACATTCGGGG	TTCTCCTCGT	CCTATAACGG	CTGCCTTGCG	AGGCAATCTC	TTGGGGTTCC	90	*	
colony sequence 11	ATGGGTAAGC	TCAGCAATCT	CCTCGTTGCA	ACATTCGGGG	TTCTCCTCGT	CCTATAACGG	CTGCCTTGCG	AGGCAATCTC	TTGGGGTTCC	90	*	
colony sequence 12	ATGGGTAAGC	TCAGCAATCT	CCTCGTTGCA	ACATTCGGGG	TTCTCCTCGT	CCTATAACGG	CTGCCTTGCG	AGGCAATCTC	TTGGGGTTCC	90	*	
colony sequence 13	ATGGTTAAGC	TCAGCAATCT	CCTCGTTGCA	ACATTCGGGG	TTCTCCTCGT	CCTATAACGG	CTGCCTTGCG	AGGCAATCTC	TTGGGGTTCC	90	*	
colony sequence 14	ATGGTTAAGC	TCAGCAATCT	CCTCGTTGCA	ACATTCGGGG	TTCTCCTCGT	C---TAACGG	CTGCCTTGCG	AGGCAATCTC	TTGGGGTTCC	87	Ŧ	G2
colony sequence 15	ATGGTTAAGC	TCAGCAATCT	CCTCGTTGCA	ACATTCGGGG	TTCTCCTCGT	CCT-TAACGG	CTGCCTTGCG	AGGCAATCTC	TTGGGGTTCC	89		
colony sequence 16	ATGGTTAAGC	TCAGCAATCT	CCTCGTTGCA	ACATTCGGGG	TTCTCCTCGT	CCT-TAACGG	CTGCCTTGCG	AGGCAATCTC	TTGGGGTTCC	89		
colony sequence 17	ATGGTTAAGC	TCAGCAATCT	CCTCGTTGCG	ACGTTCGGGG	TTCTCCTCGT	CCT-TAACGG	CTGCTTTGCG	AGGCAATCTC	TTCGGGTTCC	89		
colony sequence 18	ATGGTTAAGC	TCAGCAATCT	CCTCGTTGCG	ACGTTCGGGG	TTCTCCTCGT	CCT-TAACGG	CTGCTTTGCG	AGGCAATCTC	TTCGGGTTCC	89		G3
colony sequence 19	ATGGTTAAGC	TCAGCAATCT	CCTCGTTGCG	ACGTTCGGGG	TTCTCCTCGT	CCT-TAACGG	CTGCTTTGCG	AGGCAATCTC	TTCGGGTTCC	89		
colony sequence 20	ATGGTTAAGC	TCAGCAATCT	CCTCGTTGCG	ACGTTCGGGG	TTCTCCTCGT	CCT-TAACGG	CTGCTTTGCG	AGGCAATCTC	TTCGGGTTCC	89		

g Plant_gRNA512-1-47 (T_2 generation)

CRUC G1	ATGGGTAAGC TCAGCAATCT CCTC-GTTGC AACATTCGGG GTTCTCCTCG TCCT-TAACG GCTGCCTTGC GAGGCAATCT CTTGGGGTTC	88		
CRUC G2	ATGGTTAAGC TCAGCAATCT CCTC-GTTGC AACATTCGGG GTTCTCCTCG TCCT-TAACG GCTGCCTTGC GAGGCAATCT CTTGGGGTTC	88		
CRUC G3	ATGGTTAAGC TCAGCAATCT CCTC-GTTGC GACGTTCGGG GTTCTCCTCG TCCT-TAACG GCTGCTTTGC GAGGCAATCT CTTCGGGTTC	88		
colony sequence 1	ATGGGTAAGC TCAGCAATCT CCTC-GTTGC AACATTCGGG GTTCTCCTCG TCCT-TAACG GCTGCCTTGC GAGGCAATCT CTTGGGGTTC	88		G1
colony sequence 2	ATGGGTAAGC TCAGCAATCT CCTC-GTTGC AACATTCGGG GTTCTCCTCG TCCT-TAACG GCTGCCTTGC GAGGCAATCT CTTGGGGTTC	88		G1
colony sequence 3	ATGGGTAAGC TCAGCAATCT CCTC-GTTGC AACATTCGGG GTTCTCCTCG TCCT-TAACG GCTGCCTTGC GAGGCAATCT CTTGGGGTTC	88		G1
colony sequence 4	ATGGGTAAGC TCAGCAATCT CCTC-GTTGC AACATTCGGG GTTCTCCTCG TCCT-TAACG GCTGCCTTGC GAGGCAATCT CTTGGGGTTC	88		G1
colony sequence 5	ATGGGTAAGC TCAGCAATCT CCTC-GTTGC AACATTCGGG GTTCTCCTCG TCCT-TAACG GCTGCCTTGC GAGGCAATCT CTTGGGGTTC	88		G1
colony sequence 6	ATGGGTAAGC TCAGCAATCT CCTC-GTTGC AACATTCGGG GTTCTCCTCG TCCT-TAACG GCTGCCTTGC GAGGCAATCT CTTGGGGTTC	88		G1
colony sequence 7	ATGGGTAAGC TCAGCAATCT CCTC-GTTGC AACATTCGGG GTTCTCCTCG TCCT-TAACG GCTGCCTTGC GAGGCAATCT CTTGGGGTTC	88		G1
colony sequence 8	ATGGGTAAGC TCAGCAATCT CCTC-GTTGC AACATTCGGG GTTCTCCTCG TCCT-TAACG GCTGCCTTGC GAGGCAATCT CTTGGGGTTC	88		G1
colony sequence 9	ATGGGTAAGC TCAGCAATCT CCTC-GTTGC AACATTCGGG GTTCTCCTCG TCCT-TAACG GCTGCCTTGC GAGGCAATCT CTTGGGGTTC	88		G1
colony sequence 10	ATGGGTAAGC TCAGCAATCT CCTC-GTTGC AACATTCGGG GTTCTCCTCG TCCT-TAACG GCTGCCTTGC GAGGCAATCT CTTGGGGTTC	88		G1
colony sequence 11	ATGGGTAAGC TCAGCAATCT CCTC-GTTGC AACATTCGGG GTTCTCCTCG TCCT-TAACG GCTGCCTTGC GAGGCAATCT CTTGGGGTTC	88		G1
colony sequence 12	ATGGGTAAGC TCAGCAATCT CCTC-GTTGC AACATTCGGG GTTCTCCTCG TCCT-TAACG GCTGCCTTGC GAGGCAATCT CTTGGGGTTC	88		G1
colony sequence 13	ATGGGTAAGC TCAGCAATCT CCTC-GTTGC AACATTCGGG GTTCTCCTCG TCCT-TAACG GCTGCCTTGC GAGGCAATCT CTTGGGGTTC	88		G1
colony sequence 14	ATGGGTAAGC TCAGCAATCT CCTC-GTTGC AACATTCGGG GTTCTCCTCG TCCT-TAACG GCTGCCTTGC GAGGCAATCT CTTGGGGTTC	88		G1
colony sequence 15	ATGGGTAAGC TCAGCAATCT CCTC-GTTGC AACATTCGGG GTTCTCCTCG TCCT-TAACG GCTGCCTTGC GAGGCAATCT CTTGGGGTTC	88		G1
colony sequence 16	ATGGGTAAGC TCAGCAATCT CCTC-GTTGC AACATTCGGG GTTCTCCTCG TCCT-TAACG GCTGCCTTGC GAGGCAATCT CTTGGGGTTC	88		G1
colony sequence 17	ATGGGTAAGC TCAGCAATCT CCTC-GTTGC AACATTCGGG GTTCTCCTCG TCCT-TAACG GCTGCCTTGC GAGGCAATCT CTTGGGGTTC	88		G1
colony sequence 18	ATGGTTAAGC TCAGCAATCT CCTC-GTTGC AACATTCGGG GTTCTCCTCG TCCTATAACG GCTGCCTTGC GAGGCAATCT CTTGGGGTTC	89	*	G2
colony sequence 19	ATGGTTAAGC TCAGCAATCT CCTC-GTTGC AACATTCGGG GTTCTCCTCG TCCTATAACG GCTGCCTTGC GAGGCAATCT CTTGGGGTTC	89	*	G2
colony sequence 20	ATGGTTAAGC TCAGCAATCT CCTC-GTTGC AACATTCGGG GTTCTCCTCG TCCTATAACG GCTGCCTTGC GAGGCAATCT CTTGGGGTTC	89	*	G2
colony sequence 21	ATGGTTAAGC TCAGCAATCT CCTC-GTTGC AACATTCGGG GTTCTCCTCG TCCTATAACG GCTGCCTTGC GAGGCAATCT CTTGGGGTTC	89	*	G2
colony sequence 22	ATGGTTAAGC TCAGCAATCT CCTC-GTTGC AACATTCGGG GTTCTCCTCG TCCTATAACG GCTGCCTTGC GAGGCAATCT CTTGGGGTTC	89	*	G2
colony sequence 23	ATGGTTAAGC TCAGCAATCT CCTC-GTTGC AACATTCGGG GTTCTCCTCG TCCTATAACG GCTGCCTTGC GAGGCAATCT CTTGGGGTTC	89	*	G2
colony sequence 24	ATGGTTAAGC TCAGCAATCT CCTC-GTTGC AACATTCGGG GTTCTCCTCG TCCTTTAACG GCTGCCTTGC GAGGCAATCT CTTGGGGTTC	89	*	G2
colony sequence 25	ATGGTTAAGC TCAGCAATCT CCTC-GTTGC AACATTCGGG GTTCTCCTCG TCCT-TAACG GCTGCCTTGC GAGGCAATCT CTTGGGGTTC	88		G2
colony sequence 26	ATGGTTAAGC TCAGCAATCT CCTC-GTTGC AACATTCGGG GTTCTCCTCG TCCT-TAACG GCTGCCTTGC GAGGCAATCT CTTGGGGTTC	88		G2
colony sequence 27	ATGGTTAAGC TCACCAATCT CCTC-GTTGC AACATTCGGG GTTCTCCTCG TCCT-TAACG GCTGCCTTGC GAGGCAATCT CTTGGGGTTC	88		G2
colony sequence 29	ATGGTTAAGC TCAGCAATCT CCTC-GTTGC GACGTTCGGG GTTCTCCTCG TCCT-TAACG GCTGCTTTGC GAGGCAATCT CTTCGGGTTC	88		G3
colony sequence 30	ATGGTTAAGC TCAGCAATCT CCTC-GTTGC GACGTTCGGG GTTCTCCTCG TCCT-TAACG GCTGCTTTGC GAGGCAATCT CTTCGGGTTC	88		G3
colony sequence 31	ATGGTTAAGC TCAGCAATCT CCTC-GTTGC GACGTTCGGG GTTCTCCTCG TCCT-TAACG GCTGCTTTGC GAGGCAATCT CTTCGGGTTC	88		G3

h Plant_gRNA512-1-69 (T_2 generation)

CRUC G1	ATGGGTAAGC TCAGCAATCT CCTCGTTGCA ACATTCGGGG TTCTCCTCGT CCTTAACGGC TGCCTTGCGA GGCAATCTCT TGGGGTTCCT	90		
CRUC G2	ATGGTTAAGC TCAGCAATCT CCTCGTTGCA ACATTCGGGG TTCTCCTCGT CCTTAACGGC TGCCTTGCGA GGCAATCTCT TGGGGTTCCT	90		
CRUC G3	ATGGTTAAGC TCAGCAATCT CCTCGTTGCG ACGTTCGGGG TTCTCCTCGT CCTTAACGGC TGCTTTGCGA GGCAATCTCT TCGGGTTCCT	90		
colony sequence 1	ATGGGTAAGC TCAGCAATCT CCTCGTTGCA ACATTCGGGG TTCTCCTCGT CCTTAACGGC TGCCTTGCGA GGCAATCTCT TGGGGTTCCT	90		G1
colony sequence 2	ATGGGTAAGC TCAGCAATCT CCTCGTTGCA ACATTCGGGG TTCTCCTCGT CCTTAACGGC TGCCTTGCGA GGCAATCTCT TGGGGTTCCT	90		G1
colony sequence 3	ATGGGTAAGC TCAGCAATCT CCTCGTTGCA ACATTCGGGG TTCTCCTCGT CCTTAACGGC TGCCTTGCGA GGCAATCTCT TGGGGTTCCT	90		G1
colony sequence 4	ATGGGTAAGC TCAGCAATCT CCTCGTTGCA ACATTCGGGG TTCTCCTCGT CCTTAACGGC TGCCTTGCGA GGCAATCTCT TGGGGTTCCT	90		G1
colony sequence 5	ATGGGTAAGC TCAGCAATCT CCTCGTTGCA ACATTCGGGG TTCTCCTCGT CCT-AACGGC TGCCTTGCGA GGCAATCTCT TGGGGTTCCT	89	*	G1
colony sequence 6	ATGGGTAAGC TCAGCAATCT CCTCGTTGCA ACATTCGGGG TTCTCCTCGT CCT-AACGGC TGCCTTGCGA GGCAATCTCT TGGGGTTCCT	89	*	G1
colony sequence 7	ATGGGTAAGC TCAGCAATCT CCTCGTTGCA ACATTCGGGG TTCTCCTCGT CCT-AACGGC TGCCTTGCGA GGCAATCTCT TGGGGTTCCT	89	*	G1
colony sequence 8	ATGGGTAAGC TCAGCAATCT CCTCGTTGCA ACATTCGGGG TTCTCCTCGT CCT-AACGGC TGCCTTGCGA GGCAATCTCT TGGGGTTCCT	89	*	G1
colony sequence 9	ATGGGTAAGC TCAGCAATCT CCTCGTTGCA ACATTCGGGG TTCTCCTCGT CCTTAACGGC TGCCTTGCGA GGCAATCTCT TGGGGTTCCT	90		G1
colony sequence 10	ATGGTTAAGC TCAGCAATCT CCTCGTTGCA ACATTCGGGG TTCTCCTCGT CCTTAACGGC TGCCTTGCGA GGCAATCTCT TGGGGTTCCT	90		G2
colony sequence 11	ATGGTTAAGC TCAGCAATCT CCTCGTTGCA ACATTCGGGG TTCTCCTCGT CCTTAACGGC TGCCTTGCGA GGCAATCTCT TGGGGTTCCT	90		G2
colony sequence 12	ATGGTTAAGC TCAGCAATCT CCTCGTTGCA ACATTCGGGG TTCTCCTCGT CCTTAACGGC TGCCTTGCGA GGCAATCTCT TGGGGTTCCT	90		G2
colony sequence 13	ATGGTTAAGC TCAGCAATCT CCTCGTTGCA ACATTCGGGG TTCTCCTCGT CCTTAACGGC TGCCTTGCGA GGCAATCTCT TGGGGTTCCT	90		G2
colony sequence 14	ATGGTTAAGC TCAGCAATCT CCTCGTTGCG ACGTTCGGGG TTCTCCTCGT CCTTAACGGC TGCTTTGCGA GGCAATCTCT TCGGGTTCCT	90		G3
colony sequence 15	ATGGTTAAGC TCAGCAATCT CCTCGTTGCG ACGTTCGGGG TTCTCCTCGT CCTTAACGGC TGCTTTGCGA GGCAATCTCT TCGGGTTCCT	90		G3
colony sequence 16	ATGGTTAAGC TCAGCAATCT CCTCGTTGCG ACGTTCGGGG TTCTCCTCGT CCTTAACGGC TGCTTTGCGA GGCAATCTCT TCGGGTTCCT	90		G3
colony sequence 17	ATGGTTAAGC TCAGCAATCT CCTCGTTGCG ACGTTCGGGG TTCTCCTCGT CCTTAACGGC TGCTTTGCGA GGCAATCTCT TCGGGTTCCT	90		G3
colony sequence 18	ATGGTTAAGC TCAGCAATCT CCTCGTTGCG ACGTTCGGGG TTCTCCTCGT CCTTAACGGC TGCTTTGCGA GGCAATCTCT TCGGGTTCCT	90		G3
colony sequence 19	ATGGTTAAGC TCAGCAATCT CCTCGTTGCG ACGTTCGGGG TTCTCCTCGT CCTTAACGGC TGCTTTGCGA GGCAATCTCT TCGGGTTCCT	90		G3
colony sequence 20	ATGGTTAAGC TCAGCAATCT CCTCGTTGCG ACGTTCGGGG TTCTCCTCGT CCTTAACGGC TGCTTTGCGA GGCAATCTCT TCGGGTTCCT	90		G3
colony sequence 21	ATGGTTAAGC TCAGCAATCT CCTCGTTGCG ACGTTCGGGG TTCTCCTCGT CCTTAACGGC TGCTTTGCGA GGCAATCTCT TCGGGTTCCT	90		G3
colony sequence 22	ATGGTTAAGC TCAGCAATCT CCTCGTTGCG ACGTTCGGGG TTCTCCTCGT CCTTAACGGC TGCTTTGCGA GGCAATCTCT TCGGGTTCCT	90		G3
colony sequence 23	ATGGTTAAGC TCAGCAATCT CCTCGTTGCG ACGTTCGGGG TTCTCCTCGT CCT-AACGGC TGCTTTGCGA GGCAATCTCT TCGGGTTCCT	89	*	G3
colony sequence 24	ATGGTTAAGC TCAGCAATCT CCTCGTTGCG ACGTTCGGGG TTCTCCTCGT CCT-AACGGC TGCTTTGCGA GGCAATCTCT TCGGGTTCCT	89	*	G3

i Plant_gRNA512-1-93 (T_2 generation)

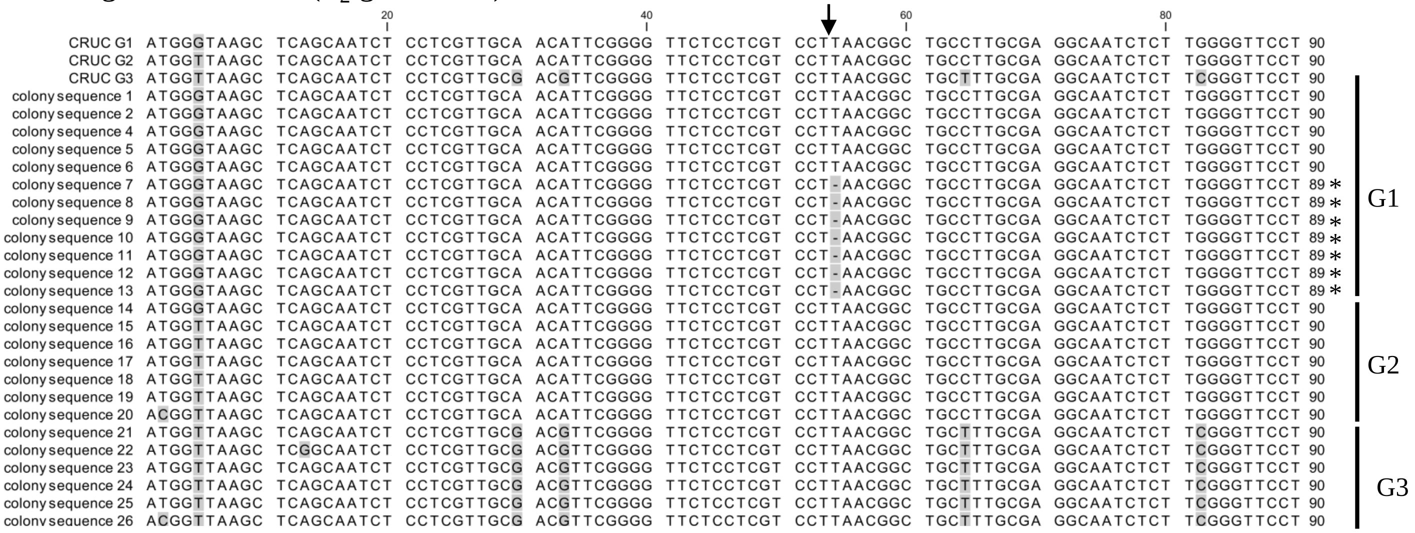

j Plant_gRNA512-1-69-8 (T_3 generation)

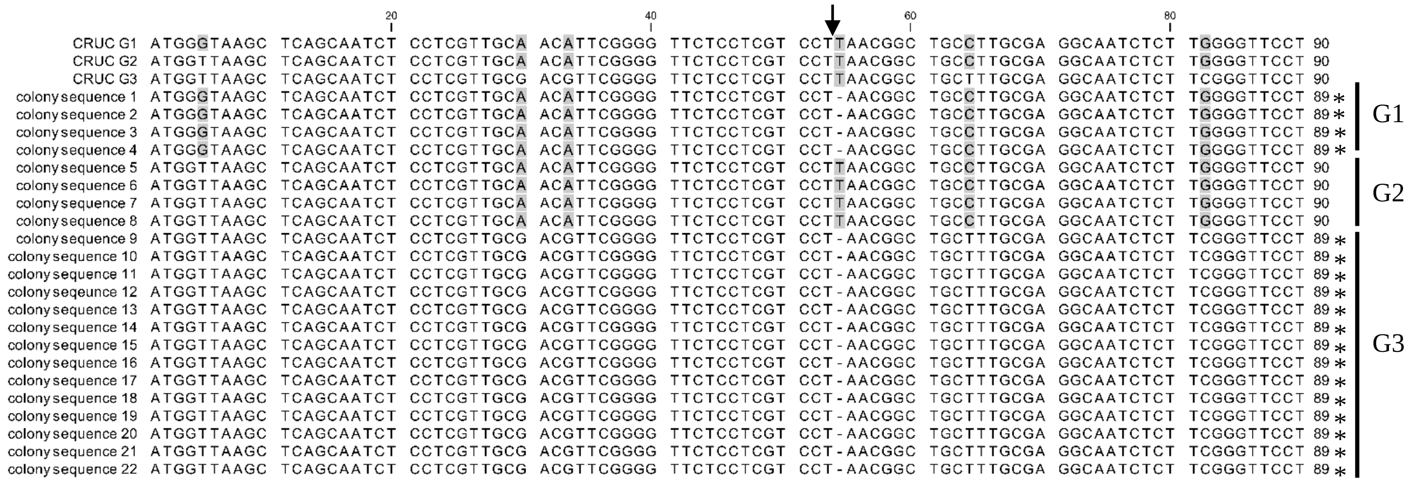

k Plant_gRNA512-1-69-8-7-11 (T_5 generation)

```
                                       20         40         60         80
CRUC G1            ATGGGTAAGC TCAGCAATCT CCTCGTTGCA ACATTCGGGG TTCTCCTCGT CCTTAACGGC TGCCTTGCGA GGCAATCTCT TGGGGTTCCT 90
CRUC G2            ATGGTTAAGC TCAGCAATCT CCTCGTTGCA ACATTCGGGG TTCTCCTCGT CCTTAACGGC TGCCTTGCGA GGCAATCTCT TGGGGTTCCT 90
CRUC G3            ATGGTTAAGC TCAGCAATCT CCTCGTTGCG ACGTTCGGGG TTCTCCTCGT CCTTAACGGC TGCTTTGCGA GGCAATCTCT TCGGGTTCCT 90
colony sequence 1  ATGGGTAAGC TCAGCAATCT CCTCGTTGCA ACATTCGGGG TTCTCCTCGT CCT-AACGGC TGCCTTGCGA GGCAATCTCT TGGGGTTCCT 89 *
colony sequence 2  ATGGGTAAGC TCAGCAATCT CCTCGTTGCA ACATTCGGGG TTCTCCTCGT CCT-AACGGC TGCCTTGCGA GGCAATCTCT TGGGGTTCCT 89 *
colony sequence 3  ATGGGTAAGC TCAGCAATCT CCTCGTTGCA ACATTCGGGG TTCTCCTCGT CCT-AACGGC TGCCTTGCGA GGCAATCTCT TGGGGTTCCT 89 *
colony sequence 4  ATGGGTAAGC TCAGCAATCT CCTCGTTGCA ACATTCGGGG TTCTCCTCGT CCT-AACGGC TGCCTTGCGA GGCAATCTCT TGGGGTTCCT 89 *
colony sequence 5  ATGGGTAAGC TCAGCAATCT CCTCGTTGCA ACATTCGGGG TTCTCCTCGT CCT-AACGGC TGCCTTGCGA GGCAATCTCT TGGGGTTCCT 89 *
colony sequence 6  ATGGGTAAGC TCAGCAATCT CCTCGTTGCA ACATTCGGGG TTCTCCTCGT CCT-AACGGC TGCCTTGCGA GGCAATCTCT TGGGGTTCCT 89 *
colony sequence 7  ATGGGTAAGC TCAGCAATCT CCTCGTTGCA ACATTCGGGG TTCTCCTCGT CCT-AACGGC TGCCTTGCGA GGCAATCTCT TGGGGTTCCT 89 *
colony sequence 8  ATGGGTAAGC TCAGCAATCT CCTCGTTGCA ACATTCGGGG TTCTCCTCGT CCT-AACGGC TGCCTTGCGA GGCAATCTCT TGGGGTTCCT 89 *
colony sequence 9  ATGGGTAAGC TCAGCAATCT CCTCGTTGCA ACATTCGGGG TTCTCCTCGT CCT-AACGGC TGCCTTGCGA GGCAATCTCT TGGGGTTCCT 89 *
colony sequence 10 ATGGGTAAGC TCAGCAATCT CCTCGTTGCA ACATTCGGGG TTCTCCTCGT CCT-AACGGC TGCCTTGCGA GGCAATCTCT TGGGGTTCCT 89 *
colony sequence 11 ATGGGTAAGC TCAGCAATCT CCTCGTTGCA ACATTCGGGG TTCTCCTCGT CCT-AACGGC TGCCTTGCGA GGCAATCTCT TGGGGTTCCT 89 *
colony sequence 12 ATGGGTAAGC TCAGCAATCT CCTCGTTGCA ACATTCGGGG TTCTCCTCGT CCT-AACGGC TGCCTTGCGA GGCAATCTCT TGGGGTTCCT 89 *
G1
colony sequence 13 ATGGTTAAGC TCAGCAATCT CCTCGTTGCA ACATTCGGGG TTCTCCTCGT CCT-AACGGC TGCCTTGCGA GGCAATCTCT TGGGGTTCCT 89 *
colony sequence 14 ATGGTTAAGC TCAGCAATCT CCTCGTTGCA ACATTCGGGG TTCTCCTCGT CCT-AACGGC TGCCTTGCGA GGCAATCTCT TGGGGTTCCT 89 *
colony sequence 15 ATGGTTAAGC TCAGCAATCT CCTCGTTGCA ACATTCGGGG TTCTCCTCGT CCT-AACGGC TGCCTTGCGA GGCAATCTCT TGGGGTTCCT 89 *
colony sequence 16 ATGGTTAAGC TCAGCAATCT CCTCGTTGCA ACATTCGGGG TTCTCCTCGT CCT-AACGGC TGCCTTGCGA GGCAATCTCT TGGGGTTCCT 89 *
colony sequence 17 ATGGTTAAGC TCAGCAATCT CCTCGTTGCA ACATTCGGGG TTCTCCTCGT CCT-AACGGC TGCCTTGCGA GGCAATCTCT TGGGGTTCCT 89 *
colony sequence 18 ATGGTTAAGC TCAGCAATCT CCTCGTTGCA ACATTCGGGG TTCTCCTCGT CCT-AACGGC TGCCTTGCGA GGCAATCTCT TGGGGTTCCT 89 *
colony sequence 19 ATGGTTAAGC TCAGCAATCT CCTCGTTGCA ACATTCGGGG TTCTCCTCGT CCT-AACGGC TGCCTTGCGA GGCAATCTCT TGGGGTTCCT 89 *
colony sequence 20 ATGGTTAAGC TCAGCAATCT CCTCGTTGCA ACATTCGGGG TTCTCCTCGT CCT-AACGGC TGCCTTGCGA GGCAATCTCT TGGGGTTCCT 89 *
colony sequence 21 ATGGTTAAGC TCAGCAATCT CCTCGTTGCA ACATTCGGGG TTCTCCTCGT CCT-AACGGC TGCCTTGCGA GGCAATCTCT TGGGGTTCCT 89 *
colony sequence 22 ATGGTTAAGC TCAGCAATCT CCTCGTTGCA ACATTCGGGG TTCTCCTCGT CCT-AACGGC TGCCTTGCGA GGCAATCTCT TGGGGTTCCT 89 *
colony sequence 23 ATGGTTAAGC TCAGCAATCT CCTCGTTGCA ACATTCGGGG TTCTCCTCGT CCT-AACGGC TGCCTTGCGA GGCAATCTCT TGGGGTTCCT 89 *
colony sequence 24 ATGGTTAAGC TCAGCAATCT CCTCGTTGCA ACATTCGGGG TTCTCCTCGT CCT-AACGGC TGCCTTGCGA GGCAATCTCT TGGGGTTCCT 89 *
colony sequence 25 ATGGTTAAGC TCAGCAATCT CCTCGTTGCA ACATTCGGGG TTCTCCTCGT CCT-AACGGC TGCCTTGCGA GGCAATCTCT TGGGGTTCCT 89 *
colony seqeunce 26 ATGGTTAAGC TCAGCAATCT CCTCGTTGCA ACATTCGGGG TTCTCCTCGT CCT-AACGGC TGCCTTGCGA GGCAATCTCT TGGGGTTCCT 89 *
colony sequence 27 ATGGTTAAGC TCAGCAATCT CCTCGTTGCA ACATTCGGGG TTCTCCTCGT CCT-AACGGC TGCCTTGCGA GGCAATCTCT TGGGGTTCCT 89 *
colony sequence 28 ATGGTTAAGC TCAGCAATCT CCTCGTTGCA ACATTCGGGG TTCTCCTCGT CCT-AACGGC TGCCTTGCGA GGCAATCTCT TGGGGTTCCT 89 *
colony sequence 29 ATGGTTAAGC TCAGCAATCT CCTCGTTGCA ACATTCGGGG TTCTCCTCGT CCT-AACGGC TGCCTTGCGA GGCAATCTCT TGGGGTTCCT 89 *
G2
colony sequence 30 ATGGTTAAGC TCAGCAATCT CCTCGTTGCG ACGTTCGGGG TTCTCCTCGT CCT-AACGGC TGCTTTGCGA GGCAATCTCT TCGGGTTCCT 89 *
colony sequence 31 ATGGTTAAGC TCAGCAATCT CCTCGTTGCG ACGTTCGGGG TTCTCCTCGT CCT-AACGGC TGCTTTGCGA GGCAATCTCT TCGGGTTCCT 89 *
colony sequence 32 ATGGTTAAGC TCAGCAATCT CCTCGTTGCG ACGTTCGGGG TTCTCCTCGT CCT-AACGGC TGCTTTGCGA GGCAATCTCT TCGGGTTCCT 89 *
colony sequence 33 ATGGTTAAGC TCAGCAATCT CCTCGTTGCG ACGTTCGGGG TTCTCCTCGT CCT-AACGGC TGCTTTGCGA GGCAATCTCT TCGGGTTCCT 89 *
colony sequence 34 ATGGTTAAGC TCAGCAATCT CCTCGTTGCG ACGTTCGGGG TTCTCCTCGT CCT-AACGGC TGCTTTGCGA GGCAATCTCT TCGGGTTCCT 89 *
colony sequence 35 ATGGTTAAGC TCAGCAATCT CCTCGTTGCG ACGTTCGGGG TTCTCCTCGT CCT-AACGGC TGCTTTGCGA GGCAATCTCT TCGGGTTCCT 89 *
colony sequence 36 ATGGTTAAGC TCAGCAATCT CCTCGTTGCG ACGTTCGGGG TTCTCCTCGT CCT-AACGGC TGCTTTGCGA GGCAATCTCT TCGGGTTCCT 89 *
G3
```

Figure S3. CRISPR-derived mutations identified in *CsCRUC*. a-k Alignment of *CsCRUC* sequences PCR amplified and cloned from plants listed in Table 1. Sequences are from independent colony clones of the *CsCRUC* amplicon generated using plant genomic DNA as template.   Cas9 cut site is indicated by an arrow. Homoeologues are indicated and assigned based on SNPs. Grouped sequences designated G1, G2 and G3 correspond to the *CsCRUC* genes from the three sub-genomes (*CsCRUC-G1*, *CsCRUC-G2*, and *CsCRUC-G3*). * indicates sequence with mutation at the Cas9 cut site, and likely represents a heritable mutation. Ŧ indicates a unique mutated sequence.
