# Supplementary material for: CRISPR/Cas9 editing of three CRUCIFERIN C homoeologues alters the seed protein profile in Camelina sativa
Source: BMC Plant Biol. 2019 Jul 4;19:292. doi: 10.1186/s12870-019-1873-0 (PMC6611024; doi:10.1186/s12870-019-1873-0)
Supplement: Supplementary file 4 — Figure S4. Electrophoresis under non-denaturing conditions demonstrates loss of CRUC in camelina is compensated by accumulation of other seed proteins. (PDF 108 kb) [file 12870_2019_1873_MOESM4_ESM.pdf]

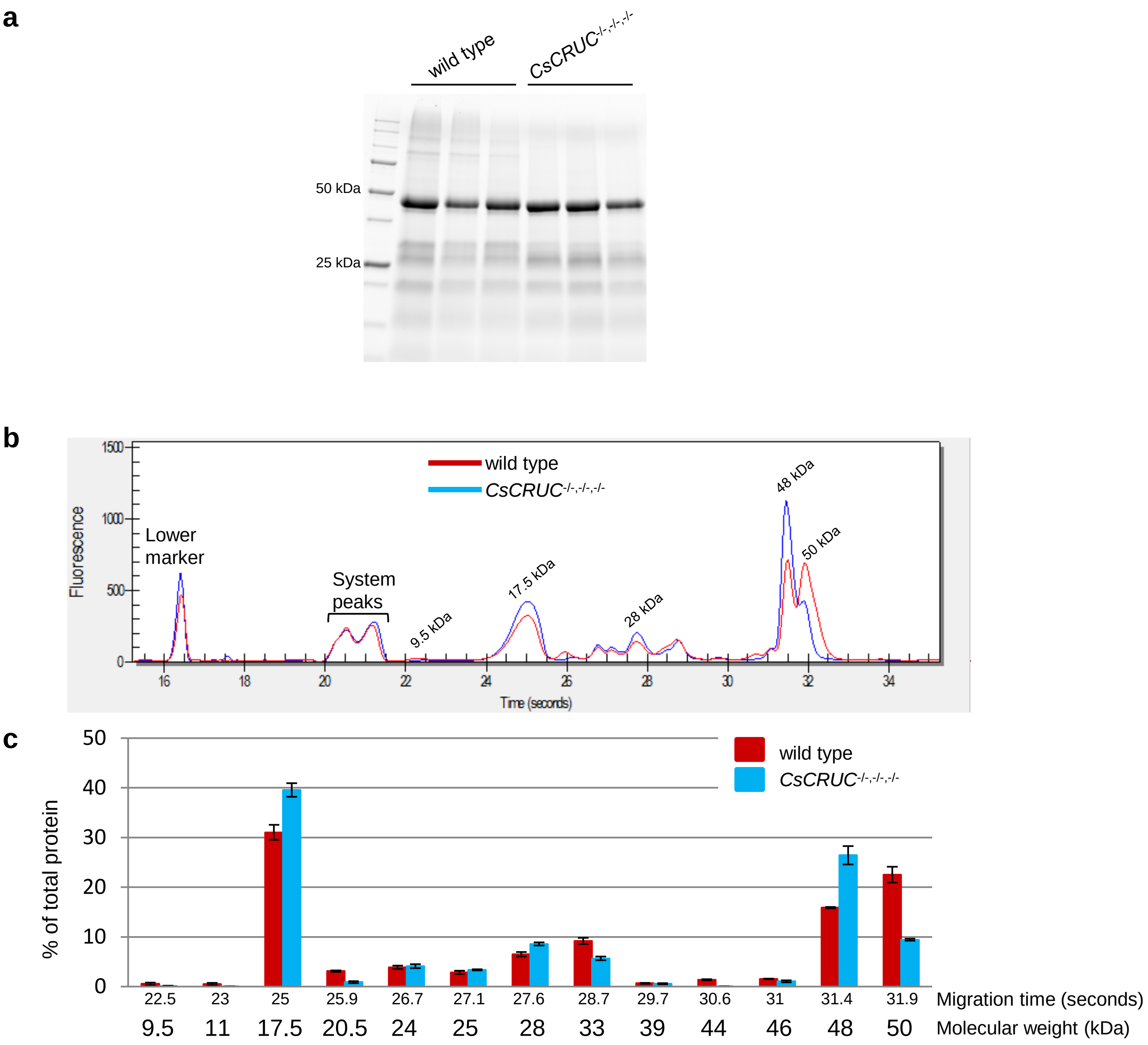


**Figure S4. Electrophoresis under non-reducing conditions demonstrates loss of CRUC in camelina is compensated by accumulation of other seed proteins.**
**a** Seed protein extracts of wild type and *CsCRUC*$^{-/-,-/-,-/-}$ electrophoresed on 8-16% acrylamide gels under non-reducing (-DTT) conditions. Gels display three biological replications. **b** Representative electropherogram from microfluidic electrophoresis of wild type and *CsCRUC*$^{-/-,-/-,-/-}$ seed protein extract under non-reducing (-DTT) conditions. Molecular weight of protein peaks are assigned based on a standard curve. **c** Quantification of **b** based on corrected peak area calculated by Experion software. Data in **c** represent the mean ± S.E.M of triplicate measurements from each of three biological replications.
