# Supplementary material for: CRISPR/Cas9 editing of three CRUCIFERIN C homoeologues alters the seed protein profile in Camelina sativa
Source: BMC Plant Biol. 2019 Jul 4;19:292. doi: 10.1186/s12870-019-1873-0 (PMC6611024; doi:10.1186/s12870-019-1873-0)
Supplement: Supplementary file 5 — Figure S5. CsCRUC β subunit has unique amino acids that may increase binding of SDS and increase relative mobility during electrophoresis. (PDF 112 kb) [file 12870_2019_1873_MOESM5_ESM.pdf]

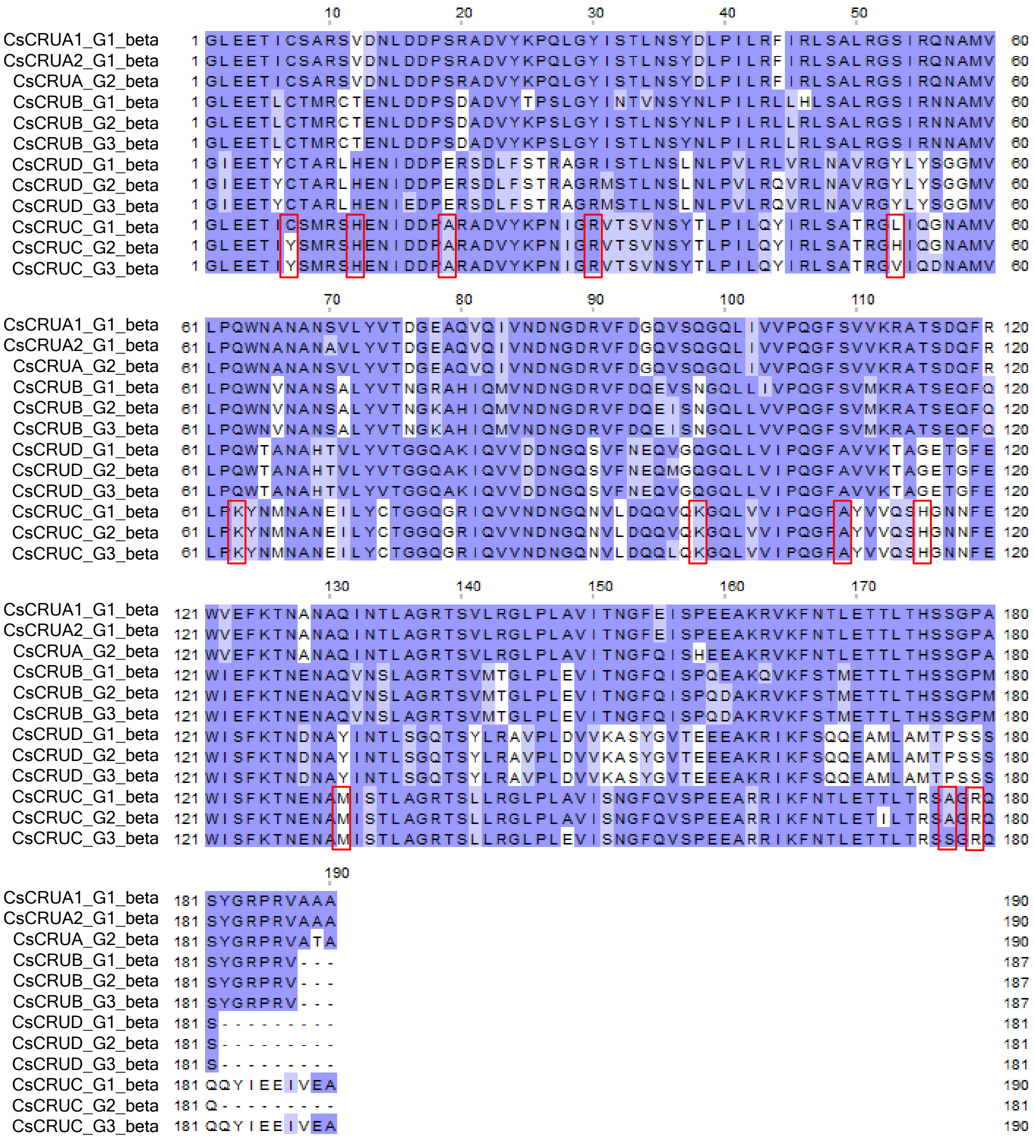

Figure S5. CsCRUC β subunit has unique amino acids that may increase binding of SDS and increase relative mobility during electrophoresis. Sequences of predicted β subunits of camelina cruciferins were aligned using Clustal Omega and coloured for similarities using the BLOSUM 62 scoring matrix. Basic and hydrophobic amino acids with increased propensity to bind SDS [48, 49] and distinct to CsCRUC β are highlighted (red box). Note that while some of these amino acids are shared with CsCRUD β, *CsCRUC* is expressed at ~20–fold greater than *CsCRUD* (Figure S1).
