# Supplementary material for: CRISPR/Cas9 editing of three CRUCIFERIN C homoeologues alters the seed protein profile in Camelina sativa
Source: BMC Plant Biol. 2019 Jul 4;19:292. doi: 10.1186/s12870-019-1873-0 (PMC6611024; doi:10.1186/s12870-019-1873-0)
Supplement: Supplementary file 6 — Figure S6. Amino acid content of cruciferin A, B, C and D in C. sativa. (PDF 70 kb) [file 12870_2019_1873_MOESM6_ESM.pdf]

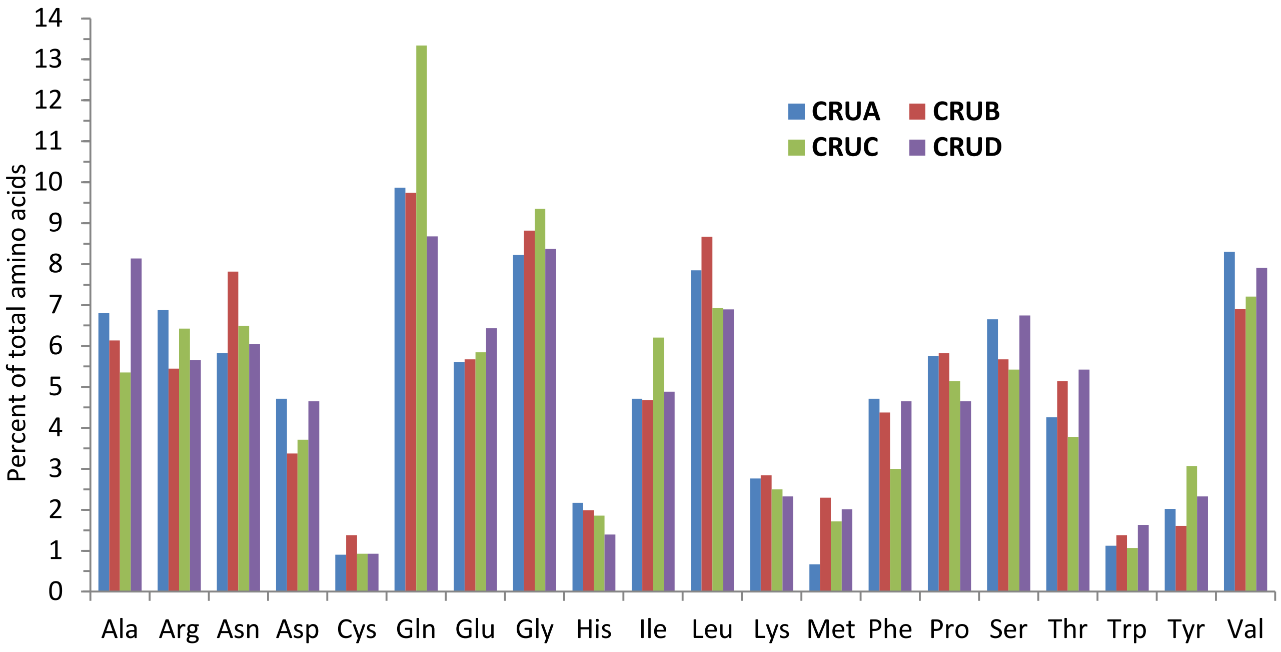

Figure S6. Amino acid content of cruciferin A, B, C and D in *C. sativa*. Values are based on translated nucleotide sequence of gene region not including the predicted signal peptide, and represent average of the three homoeologues for each cruciferin class.
