# Supplementary material for: CRISPR/Cas9 editing of three CRUCIFERIN C homoeologues alters the seed protein profile in Camelina sativa
Source: BMC Plant Biol. 2019 Jul 4;19:292. doi: 10.1186/s12870-019-1873-0 (PMC6611024; doi:10.1186/s12870-019-1873-0)
Supplement: Supplementary file 7 — Table S1. Translated protein sequence resulting from premature stop codon in CsCRUC knockout alleles. (DOCX 22 kb) [file 12870_2019_1873_MOESM7_ESM.docx]

**Additional file 7: Table S1.** Translated protein sequence resulting from premature stop codon in *CsCRUC* knockout alleles.

| plant | allele | InDel | Gene sequence^†^ |
| --- | --- | --- | --- |
| plant_gRNA512-1-69-8-7-11 | *CsCRUC_G1 -/-* | -T | GGGTTCTCCTCGTCC-TAACGGCTGC**C**TTGCGAGGCAATCTCTT**G**GGGTTCCTCCTCA**A**CTAC AGAACGAGTGTAACCTTGATAACC*TAG* |
|  | *CsCRUC_G2 -/-* | -T | GGGTTCTCCTCGTCC-TAACGGCTGC**C**TTGCGAGGCAATCTCTT**G**GGGTTCCTCCTCA**GC**TAC AGAACGAGTGTAACCTTGATAACC*TAG* |
|  | *CsCRUC_G3 -/-* | -T | GGGTTCTCCTCGTCC-TAACGGCTGC**T**TTGCGAGGCAATCTCTT**C**GGGTTCCTCCTCA**GT**TAC AGAACGAGTGTAACCTTGATAACC*TAG* |
|  |  |  | Protein sequence (* indicates stop codon) |
|  | *CsCRUC_G1 -/-* |  | MGKLSNLLVATFGVLLVLTAALRGNLLGFLLNYRTSVTLIT* |
|  | *CsCRUC_G2 -/-* |  | MVKLSNLLVATFGVLLVLTAALRGNLLGFLLSYRTSVTLIT* |
|  | *CsCRUC_G3 -/-* |  | MVKLSNLLVATFGVLLVLTAALRGNLFGFLLSYRTSVTLIT* |

^†^The Cas9 cut site is highlighted in grey. PAM sequences are underlined and SNPs that distinguish homoeologues are bolded. Nucleotide deletions are indicated ( – ). Stop codon is italicised.
