# Supplementary material for: CRISPR/Cas9 editing of three CRUCIFERIN C homoeologues alters the seed protein profile in Camelina sativa
Source: BMC Plant Biol. 2019 Jul 4;19:292. doi: 10.1186/s12870-019-1873-0 (PMC6611024; doi:10.1186/s12870-019-1873-0)
Supplement: Supplementary file 8 — Table S2. Predicted molecular weight of camelina cruciferins and derived α and β chains. (DOCX 22 kb) [file 12870_2019_1873_MOESM8_ESM.docx]

**Additional file 8: Table S2.** Predicted molecular weight of camelina cruciferins and derived α and β chains.

| Gene | Predicted Mw (kDa) | | |
| --- | --- | --- | --- |
|  | Unprocessed | α chain | β chain |
| CsCRUA1-G1 Csa11g070580  CsCRUA2-G1 Csa11g070590  CsCRUA - G2 Csa18g009670 | 52.02  52.03  51.95 | 28.57  28.59  28.64 | 20.78  20.77  20.85 |
| CsCRUB - G1 Csa17g006950  CsCRUB - G2 Csa14g004960  CsCRUB - G3 Csa03g005050 | 50.74  50.49  50.57 | 27.41  27.14  27.19 | 20.75  20.77  20.77 |
| CsCRUC - G1 Csa11g015240  CsCRUC - G2 Csa10g014100  CsCRUC - G3 Csa12g021990 | 54.86  53.95  55.39 | 31.43  31.46  31.70 | 21.11  20.14  21.31 |
| CsCRUD - G1 Csa17g006960  CsCRUD - G2 Csa14g004970  CsCRUD - G3 Csa03g005060 | 50.46  50.12  50.04 | 27.91  27.51  27.45 | 19.81  19.87  19.85 |
