# Supplementary material for: CRISPR/Cas9 editing of three CRUCIFERIN C homoeologues alters the seed protein profile in Camelina sativa
Source: BMC Plant Biol. 2019 Jul 4;19:292. doi: 10.1186/s12870-019-1873-0 (PMC6611024; doi:10.1186/s12870-019-1873-0)
Supplement: Supplementary file 9 — Table S3. Predicted total molecular weight of camelina napins and derived small and large subunits. (DOCX 22 kb) [file 12870_2019_1873_MOESM9_ESM.docx]

**Additional file 9: Table S3.** Predicted total molecular weight of camelina napins and derived small and large subunits.

|  | Predicted Mw (kDa) | | |
| --- | --- | --- | --- |
| Gene | Unprocessed | Small subunit | Large subunit |
| Cs2S1-G1 Csa11g017020 | 18.83 | 4.16 | 9.24 |
| Cs2S1-G3 Csa12g024735 | 19.01 | 4.18 | 9.31 |
| Cs2S2-G1 Csa11g017010 | 18.25 | 4.05 | 8.91 |
| Cs2S2-G3 Csa12g024731 | 18.46 | 4.17 | 8.89 |
| Cs2S3-G1 Csa11g017005 | 18.86 | 4.36 | 8.45 |
| Cs2S3-G3 Csa12g024725 | 18.87 | 4.36 | 8.45 |
| Cs2S4-G1 Csa11g017001 | 18.83 | 4.18 | 9.22 |
| Cs2S4-G3 Csa12g024721 | 18.93 | 4.18 | 9.27 |
