# Supplementary material for: CRISPR/Cas9 editing of three CRUCIFERIN C homoeologues alters the seed protein profile in Camelina sativa
Source: BMC Plant Biol. 2019 Jul 4;19:292. doi: 10.1186/s12870-019-1873-0 (PMC6611024; doi:10.1186/s12870-019-1873-0)
Supplement: Supplementary file 10 — Table S4. Amino acid content of camelina cruciferins and napins. (DOCX 23 kb) [file 12870_2019_1873_MOESM10_ESM.docx]

**Additional file 10: Table S4.** Amino acid content of camelina cruciferins and napins.

| Amino Acid | Average amino acid content^*^ (% residue frequency in mature protein) | |
| --- | --- | --- |
|  | CsCRU | Cs2S |
| A Ala | 6.61 | 5.43 |
| C Cys | 1.03 | 6.97 |
| D Asp | 4.11 | 2.61 |
| E Glu | 5.89 | 3.15 |
| F Phe | 4.18 | 2.40 |
| G Gly | 8.69 | 6.31 |
| H His | 1.85 | 1.85 |
| I Ile | 5.12 | 3.16 |
| K Lys | 2.61 | 1.64 |
| L Leu | 7.59 | 6.10 |
| M Met | 1.67 | 1.96 |
| N Asn | 6.55 | 2.29 |
| P Pro | 5.34 | 9.37 |
| Q Gln | 10.41 | 22.19 |
| R Arg | 6.21 | 11.52 |
| S Ser | 6.12 | 4.78 |
| T Thr | 4.65 | 2.07 |
| V Val | 7.58 | 5.12 |
| W Trp | 1.30 | 0.22 |
| Y Tyr | 2.26 | 0.87 |

^*^Mean value of all homoelogues of gene family members for respective mature proteins based on predicted processing sites leading to removal of signal peptide in cruciferins and napins and proteolytic processing of the napin proprotein.
