# Supplementary material for: CRISPR/Cas9 editing of three CRUCIFERIN C homoeologues alters the seed protein profile in Camelina sativa
Source: BMC Plant Biol. 2019 Jul 4;19:292. doi: 10.1186/s12870-019-1873-0 (PMC6611024; doi:10.1186/s12870-019-1873-0)
Supplement: Supplementary file 11 — Table S5. Fatty acid profile of CsCRUC knockout seeds. (DOCX 13 kb) [file 12870_2019_1873_MOESM11_ESM.docx]

**Additional file 11: Table S5.** Fatty acid profile of *CsCRUC* knockout seeds (% total fatty acids)

| Fatty acid | Wild Type | *CsCRUC*^-/-,-/-,-/-^ |
| --- | --- | --- |
| 16:0 | 5.42 ± 0.10 | 5.64 ± 0.08** |
| 16:1 | 0.15 ± 0.01 | 0.15 ± 0.01 |
| 18:0 | 2.28 ± 0.09 | 3.05 ± 0.07*** |
| 18:1^†^ | 12.85 ± 0.48 | 12.09 ± 0.80 |
| 18:2 | 17.50 ± 0.36 | 17.32 ± 0.61 |
| 18:3 | 37.25 ± 0.63 | 35.76 ± 1.02* |
| 20:0 | 1.77 ± 0.10 | 2.54 ± 0.10*** |
| 20:1 | 14.71 ± 0.08 | 14.60 ± 0.13 |
| 20:2 | 2.11 ± 0.03 | 2.28 ± 0.03*** |
| 22:0 | 0.41 ± 0.01 | 0.56 ± 0.02*** |
| 22:1 | 4.19 ± 0.20 | 4.60 ± 0.21* |
| 22:2 | 0.23 ± 0.01 | 0.26 ± 0.02* |
| 24:0 | 0.28 ± 0.02 | 0.31 ± 0.01* |
| 24:1 | 0.79 ± 0.03 | 0.78 ± 0.04 |

Values represent means ± S.D. for seed samples from five biological replicates.
Significance of differences between means assessed by Student’s t-test: *, p<0.05; **, p<0.01; ***, p<0.001.
^†^Represents content of delta 9 and delta 11 isomers.
