# Supplementary material for: CRISPR/Cas9 editing of three CRUCIFERIN C homoeologues alters the seed protein profile in Camelina sativa
Source: BMC Plant Biol. 2019 Jul 4;19:292. doi: 10.1186/s12870-019-1873-0 (PMC6611024; doi:10.1186/s12870-019-1873-0)
Supplement: Supplementary file 12 — Table S6. Primer, probe and oligonucleotide sequences. (DOCX 23 kb) [file 12870_2019_1873_MOESM12_ESM.docx]

**Additional file 12: Table S6.** Primer, probe and oligonucleotide sequences.

| **Sequencing and cloning primers^1^** | |
| --- | --- |
| *CsCRUC* sequencing primer  (all homoeologues) | CACAGACTGACGTGTAA |
| *CsCRUC* reverse primer  (all homoeologues) | TATCtctagaCCTTGAACAACGTAGGAGAT |
| *CsCRUC* forward primer  (all homoeologues) | TATGccatggAGAGACACTTGCTCGAGC |
| EF1-prom-5'KpnNheMfe | aaatggtaccgctagccaattGCAACACGGTGGTCCATATC |
| EF1-prom-3'AvrClaSna | atccctaggatcgattacgtaATAGCTGTCAAAACAAAAACAAAAATCGAAACATC |
| Cas9At_S4 | GACGACTCACTCACGTTCAAAG |
| Cas9At_AS2 | AGCGTTAAGCAACTGTCTCC |
| **Droplet digital PCR probes and primers** | |
| *CsCRUC* reference probe  (Note: used for both gRNA512 and gRNA510) | CGGTCTCTACTTGCCCACCTTCTTCA |
| *CsCRUC* drop-off probe  (Note: used for gRNA512) | TCCTCGTCCTTAACGGCTGC |
| *CsCRUC* drop-off probe  (Note: used for gRNA510) | CACTTTTGATAGTTTCGGTGGC |
| *CsCRUC* forward primer  (Note: used for gRNA512) | CTCAGCAATCTCCTCGTTG |
| *CsCRUC* forward primer  (Note: used for gRNA510) | TACAGAACGAGTGTAACCT |
| *CsCRUC* reverse primer  (Note: used for both gRNA512 and gRNA510) | ACCTTGAACAACGTAGGAGAT |
| *Cas9* probe | CGCCACTCTATCAAGAAGAACCTCATCG |
| *Cas9* forward primer | CCTCCAAGAAATTCAAGGTCCTA |
| *Cas9* reverse primer | CAATCTAGTAGCCTCAGCAGTC |
| *CsPDF2* probe | ATCTCGGATCCCATTACTGGCGC |
| *CsPDF2* forward primer | TTCGTGCAGTATCGCTTCTT |
| *CsPDF2* reverse primer | GATGTTTGGAACTCTGTCTTTAGATG |
| **Oligonucleotides for generating gRNA cassettes^2^** | |
| gRNA512_CsCRUC_Ex1_TS1_20_U6p_S | attGGGGTTCTCCTCGTCCTTAA |
| gRNA512_CsCRUC_Ex1_TS1_20_U6p_AS | aaacTTAAGGACGAGGAGAACCC |
| gRNA510_CsCRUC_Ex1_NT1_20_U6p_S | attGGCCTCACTTTTGATAGTTT |
| gRNA510_CsCRUC_Ex1_NT1_20_U6p_AS | aaacAAACTATCAAAAGTGAGGC |

^1^Restriction enzyme sites in lower case.
^2^Overhang compatible with BsaI sites in pET28+AtU6-26_gRNA_attR4-L2 are in lower case.
