# Supplementary material for: CRISPR/Cas9 editing of three CRUCIFERIN C homoeologues alters the seed protein profile in Camelina sativa
Source: BMC Plant Biol. 2019 Jul 4;19:292. doi: 10.1186/s12870-019-1873-0 (PMC6611024; doi:10.1186/s12870-019-1873-0)
Supplement: Supplementary file 13 — DNA sequence of gRNA expression cassette. (DOCX 25 kb) [file 12870_2019_1873_MOESM13_ESM.docx]

**Additional file 13** DNA sequence of gRNA expression cassette, including *AtU6-26* regulatory regions (green), *lacZα* cassette (blue) for GoldenGate cloning of annealed oligos encoding unique gRNA spacer sequences, and gRNA scaffold (red). BsaI recognition sites are italicised.

ACTTTCCATTCGGAGTTTTTGTATCTTGTTTCATAGTTTGTCCCAGGATTAGAATGATTA

GGCATCGAACCTTCAAGAATTTGATTGAATAAAACATCTTCATTCTTAAGATATGAAGAT

AATCTTCAAAAGGCCCCTGGGAATCTGAAAGAAGAGAAGCAGGCCCATTTATATGGGAAA

GAACAATAGTATTTCTTATATAGGCCCATTTAAGTTGAAAACAATCTTCAAAAGTCCCAC

ATCGCTTAGATAAGAAAACGAAGCTGAGTTTATATACAGCTAGAGTCGAAGTAGTGATTG

G***GAGACC***GCACGTGTTGACAATTAATCATCGGCATAGTATATCGGCATAGTATAATACGA

CAAGGTGAGGAACTAACTCATGACCATGATTACGGATTCACTGGCCGTCGTTTTACAACG

TCGTGACTGGGAAAACCCTGGCGTTACCCAACTTAATCGCCTTGCAGCACATCCCCCTTT

CGCCAGCTGGCGTAATAGCGAAGAGGCCCGCACCGATCGCCCTTCCCAACAGTTGCGCAG

CCTGAATGGCGAATGGCGCTTTGCCTGGTTTCCGGCACCAGAAGCGGTGCCGGAAAGCTG

GCTGGAGTGCGATCTTCCTGAGGCCGATACGTAAGCCTAGGCCAAAGCCCGCCGAAAGGC

GGGCTTTTCTGTATCTGGACTAGTA***GGTCTC***AGTTTTAGAGCTAGAAATAGCAAGTTAAA

ATAAGGCTAGTCCGTTATCAACTTGAAAAAGTGGCACCGAGTCGGTGCTTTTTTTTTGCA

AAATTTTCCAGATCGATTTCTTCTTCCTCTGTTCTTCGGCGTTCAATTTCTGGGTTTTTC

TCTTCGTTTTCTGTAACTGAAT
