# Supplementary material for: CRISPR/Cas9 editing of three CRUCIFERIN C homoeologues alters the seed protein profile in Camelina sativa
Source: BMC Plant Biol. 2019 Jul 4;19:292. doi: 10.1186/s12870-019-1873-0 (PMC6611024; doi:10.1186/s12870-019-1873-0)
Supplement: Supplementary file 14 — DNA sequence of Cas9optAt optimised for general expression in crucifers. (DOCX 27 kb) [file 12870_2019_1873_MOESM14_ESM.docx]

**Additional file 14** DNA sequence of *Cas9optAt* optimised for general expression in crucifers by considering *Arabidopsis thaliana* codon usage, including nuclear localisation sequence from SV40 large T-antigen (red), 3xFLAG epitope tag (blue), nuclear localisation sequence from nucleoplasmin (green) and *S. pyrognenes* Cas9 (purple).

ATGGCTCCTAAGAAAAAGCGTAAAGTGGGTGGCAGTGGAGGAGATTACAAAGATCACGAT

GGGGATTACAAAGACCATGACATCGACTACAAGGACGATGATGACAAAGGTGGTAGCGCT

GGGTCTGGAGCTGCTGATAAGAAATACTCTATCGGACTCGATATTGGAACGAATTCAGTA

GGGTGGGCTGTGATCACCGACGAATACAAAGTACCCTCCAAGAAATTCAAGGTCCTAGGA

AATACTGATCGCCACTCTATCAAGAAGAACCTCATCGGAGCATTACTTTTTGACTCTGGC

GAGACTGCTGAGGCTACTAGATTGAAGAGGACCGCACGTAGAAGATACACCCGAAGGAAA

AACAGAATATGCTATTTGCAAGAGATTTTCAGCAATGAGATGGCTAAAGTTGACGATTCT

TTCTTCCATCGACTTGAAGAGTCATTCCTAGTTGAGGAGGACAAAAAGCATGAACGGCAT

CCGATTTTTGGGAATATAGTGGATGAAGTTGCTTATCACGAAAAGTATCCCACGATCTAC

CACCTCCGTAAAAAGTTGGTGGATAGTACCGATAAAGCGGATCTCAGACTCATATATCTG

GCTCTTGCTCACATGATTAAGTTTCGTGGACACTTCCTCATAGAAGGAGATCTTAACCCA

GATAATAGCGACGTTGATAAGTTGTTTATACAATTGGTGCAAACGTACAACCAGCTTTTC

GAGGAAAATCCTATTAACGCCAGTGGAGTCGATGCCAAAGCAATTCTGTCAGCAAGATTG

TCTAAGTCAAGACGCCTTGAAAATCTAATCGCCCAATTGCCAGGTGAAAAGAAGAACGGA

TTGTTCGGCAATCTTATTGCACTTTCGCTTGGTTTGACGCCGAACTTTAAGTCAAACTTC

GATTTGGCGGAGGATGCTAAACTTCAGTTATCCAAAGACACTTATGACGATGATCTCGAC

AATCTGCTTGCCCAAATTGGAGATCAATATGCAGACCTGTTCCTGGCTGCTAAAAACTTG

AGTGATGCTATCCTGCTCAGTGACATCCTCAGAGTTAATACTGAGATCACTAAAGCACCA

CTCTCAGCAAGTATGATAAAGAGATACGATGAGCATCATCAAGACCTTACACTCTTGAAG

GCTCTTGTAAGGCAACAGCTACCAGAGAAGTATAAGGAAATCTTTTTCGATCAATCCAAA

AACGGTTACGCTGGATACATCGACGGGGGAGCATCTCAAGAGGAGTTCTACAAGTTCATC

AAGCCAATCCTTGAGAAAATGGATGGGACTGAAGAGTTATTGGTGAAGCTCAACAGAGAG

GATCTTCTAAGGAAACAGAGGACCTTTGATAATGGATCAATCCCACACCAAATCCATTTG

GGTGAATTACATGCCATTTTGCGCCGTCAGGAGGATTTCTACCCTTTCTTAAAAGATAAC

AGAGAGAAAATTGAAAAGATTTTGACTTTCCGTATTCCGTATTACGTTGGACCCCTAGCA

AGGGGTAACTCCCGATTTGCATGGATGACCAGGAAGTCTGAGGAAACGATTACACCTTGG

AATTTTGAGGAAGTAGTCGATAAAGGCGCTTCTGCACAGTCTTTCATCGAGCGAATGACA

AACTTCGACAAAAATCTGCCCAATGAGAAGGTGCTTCCTAAGCACTCTTTACTCTATGAA

TACTTTACCGTTTACAACGAGCTTACAAAGGTGAAGTACGTCACAGAGGGTATGCGGAAA

CCTGCTTTCCTTTCTGGCGAACAGAAAAAGGCGATTGTTGATTTGTTGTTCAAGACTAAT

CGGAAAGTGACAGTTAAACAACTTAAAGAAGATTACTTCAAAAAGATCGAATGTTTTGAC

TCAGTGGAAATATCAGGTGTAGAGGATCGTTTCAATGCGTCTCTTGGGACTTATCACGAT

TTGCTGAAGATCATTAAGGATAAGGACTTTTTAGACAATGAGGAAAATGAGGACATCCTC

GAAGATATTGTATTGACTCTCACGCTATTTGAAGATCGAGAGATGATCGAGGAGAGGCTT

AAAACGTATGCACATCTTTTCGATGACAAGGTGATGAAGCAACTGAAACGACGCAGATAT

ACCGGTTGGGGAAGGCTCTCTCGGAAGTTGATCAATGGAATACGTGATAAGCAGTCTGGA

AAGACAATCCTCGACTTTTTGAAAAGTGATGGATTTGCTAATCGAAATTTCATGCAACTT

ATTCACGACGACTCACTCACGTTCAAAGAGGACATTCAAAAGGCACAAGTATCAGGGCAG

GGAGATTCCCTCCATGAACATATTGCAAATCTGGCCGGTTCTCCCGCAATTAAGAAAGGC

ATACTTCAAACAGTCAAAGTGGTCGATGAATTAGTTAAAGTAATGGGTCGTCATAAGCCA

GAGAATATTGTTATTGAAATGGCTAGGGAAAACCAGACCACTCAGAAAGGACAGAAAAAC

TCAAGAGAAAGGATGAAACGAATCGAGGAGGGAATCAAGGAGCTTGGTAGCCAAATCTTA

AAAGAGCATCCTGTCGAGAATACCCAACTTCAAAACGAGAAATTGTATCTTTACTACCTC

CAAAATGGTAGGGATATGTATGTAGATCAGGAATTAGACATTAATCGGCTCTCGGATTAT

GATGTTGACCATATCGTTCCTCAGTCTTTCCTTAAAGATGATTCGATAGACAATAAGGTC

CTGACTAGATCCGATAAGAACAGAGGAAAGAGTGATAATGTTCCCAGTGAGGAAGTTGTT

AAGAAGATGAAAAACTACTGGAGACAGTTGCTTAACGCTAAACTGATTACTCAAAGGAAA

TTTGATAACCTCACTAAGGCTGAACGAGGTGGACTGAGCGAATTGGATAAAGCTGGATTC

ATCAAAAGACAATTGGTCGAGACAAGGCAGATTACCAAGCATGTGGCACAAATACTTGAT

TCAAGGATGAATACCAAATATGATGAGAATGATAAACTTATTAGAGAGGTTAAAGTAATT

ACACTGAAATCTAAGTTGGTGTCTGATTTCCGTAAGGATTTCCAGTTTTACAAAGTGCGA

GAAATCAATAACTACCACCACGCGCACGACGCATATCTCAACGCTGTTGTCGGGACAGCC

CTCATTAAGAAGTATCCTAAGCTCGAATCAGAGTTCGTTTATGGTGATTACAAAGTTTAT

GATGTCCGCAAAATGATTGCAAAATCAGAGCAAGAGATTGGTAAAGCGACAGCCAAATAC

TTTTTCTATTCTAACATTATGAACTTTTTCAAGACTGAAATAACTCTGGCGAATGGGGAA

ATTCGTAAGAGGCCTTTGATTGAGACAAACGGTGAGACTGGAGAGATAGTATGGGACAAA

GGCCGCGATTTTGCTACTGTTAGGAAGGTTTTGAGTATGCCGCAAGTAAACATCGTTAAG

AAAACAGAAGTTCAGACTGGAGGTTTTAGTAAGGAGAGCATCCTGCCAAAGAGGAACTCC

GATAAGCTCATCGCTCGTAAAAAGGATTGGGACCCGAAAAAGTATGGCGGTTTCGACTCT

CCTACTGTTGCCTATAGCGTACTTGTCGTGGCCAAGGTCGAGAAAGGAAAAAGCAAAAAG

CTCAAGAGCGTTAAGGAACTCCTTGGTATCACTATAATGGAAAGATCGTCATTCGAGAAA

AACCCGATAGACTTCCTAGAAGCTAAAGGGTATAAAGAGGTCAAAAAGGATCTCATTATC

AAACTGCCTAAGTATTCGCTATTTGAATTGGAGAATGGTAGAAAGAGAATGCTTGCAAGT

GCTGGAGAACTTCAGAAGGGAAATGAGCTCGCTTTGCCGTCAAAATACGTGAATTTCCTT

TATCTCGCTTCACATTATGAGAAATTGAAAGGTTCACCAGAGGATAACGAGCAGAAACAG

TTATTTGTGGAACAACACAAACATTACCTCGATGAGATAATAGAGCAGATAAGCGAGTTT

TCGAAGAGGGTGATTTTGGCTGATGCAAATCTCGATAAAGTGCTCTCGGCATATAACAAG

CATAGAGATAAGCCTATAAGGGAGCAAGCGGAGAACATTATTCACCTTTTTACCCTAACA

AACTTGGGCGCACCAGCCGCTTTTAAGTACTTTGACACAACTATCGACCGAAAAAGATAC

ACAAGTACCAAAGAGGTCTTGGACGCTACTTTAATCCATCAATCCATCACGGGACTTTAT

GAAACCAGAATTGATTTGAGCCAGTTAGGAGGAGATGGCGCCGGTTCTGGTACCGGCAAA

CGTCCAGCAGCGACTAAAAAGGCGGGTCAGGCCAAAAAGAAGAAATGA
