# Supplementary material for: CRISPR/Cas9 editing of three CRUCIFERIN C homoeologues alters the seed protein profile in Camelina sativa
Source: BMC Plant Biol. 2019 Jul 4;19:292. doi: 10.1186/s12870-019-1873-0 (PMC6611024; doi:10.1186/s12870-019-1873-0)
Supplement: Supplementary file 15 — DNA sequences of synthesised cassettes used to validate drop-off assays. (DOCX 25 kb) [file 12870_2019_1873_MOESM15_ESM.docx]

**Additional file 15** DNA sequences of synthesised cassettes used to validate drop-off assays, including wild type *CsCRUC* and *CsCRUC^Δ4^* encoding four base pair deletion at the predicted Cas9 cut site for either gRNA510 or gRNA512.
*CsCRUC*:
GAATTCACAAAAAGAAAATGGTTAAGCTCAGCAATCTCCTCGTTGCAACATTCGGGGTTC
TCCTCGTCCTTAACGGCTGCCTTGCGAGGCAATCTCTTGGGGTTCCTCCTCAGCTACAGA
ACGAGTGTAACCTTGATAACCTAGACGTTCTTGAAGCCACCGAAACTATCAAAAGTGAGG
CCGGTCAGATCGAGTACTGGGATCACAACCACCCTCACCTCCGATGTGCTGGTGTTTCCG
TAGCTCGTTATGTAATTGAACAAGGCGGTCTCTACTTGCCCACCTTCTTCACTTCCCCAA
AAATCTCCTACGTTGTTCAAGGTCTATATATCAATCTCTCCAGAATTC

*CsCRUC^Δ4^*_gRNA510
GAATTCACAAAAAGAAAATGGTTAAGCTCAGCAATCTCCTCGTTGCAACATTCGGGGTTC
TCCTCGTCCTTAACGGCTGCCTTGCGAGGCAATCTCTTGGGGTTCCTCCTCAGCTACAGA
ACGAGTGTAACCTTGATAACCTAGACGTTCTTGAAGCCACCGAATCAAAAGTGAGGCCGG
TCAGATCGAGTACTGGGATCACAACCACCCTCACCTCCGATGTGCTGGTGTTTCCGTAGC
TCGTTATGTAATTGAACAAGGCGGTCTCTACTTGCCCACCTTCTTCACTTCCCCAAAAAT
CTCCTACGTTGTTCAAGGTCTATATATCAATCTCTCCAGAATTC

*CsCRUC^Δ4^*_gRNA512

GAATTCACAAAAAGAAAATGGTTAAGCTCAGCAATCTCCTCGTTGCAACATTCGGGGTTC

TCCTCGTCACGGCTGCCTTGCGAGGCAATCTCTTGGGGTTCCTCCTCAGCTACAGAACGA

GTGTAACCTTGATAACCTAGACGTTCTTGAAGCCACCGAAACTATCAAAAGTGAGGCCGG

TCAGATCGAGTACTGGGATCACAACCACCCTCACCTCCGATGTGCTGGTGTTTCCGTAGC

TCGTTATGTAATTGAACAAGGCGGTCTCTACTTGCCCACCTTCTTCACTTCCCCAAAAAT

CTCCTACGTTGTTCAAGGTCTATATATCAATCTCTCCAGAATTC
